# Moderately hypo-fractionated radiotherapy combined with S-1 in inoperable locally advanced esophageal squamous cell carcinoma: A prospective, single-arm phase II study (GASTO-1045)

**DOI:** 10.3389/fonc.2023.1138304

**Published:** 2023-03-10

**Authors:** Rui Zhou, Guangyu Luo, Suping Guo, Yingjia Wu, Qiaoting Luo, Daquan Wang, Naibin Chen, Fangjie Liu, Jinyu Guo, Wenfeng Ye, Bo Qiu, Hui Liu

**Affiliations:** ^1^ Department of Radiation Oncology, Sun Yat-sen University Cancer Center, Guangzhou, China; ^2^ State Key Laboratory of Oncology in South China, Guangzhou, China; ^3^ Collaborative Innovation Center for Cancer Medicine, Sun Yat-sen University Cancer Center, Guangzhou, China; ^4^ Lung Cancer Institute of Sun Yat-sen University, Guangzhou, China; ^5^ Guangdong Association Study of Thoracic Oncology, Guangzhou, China; ^6^ Department of Endoscopy and Laser, Sun Yat-sen University Cancer Center, Guangzhou, China; ^7^ Department of Nutrition, Sun Yat-sen University Cancer Center, Guangzhou, China

**Keywords:** locally advanced esophageal squamous cell carcinoma, moderately hypo-fractionated radiotherapy, S-1, percutaneous endoscopic gastrostomy, concurrent chemoradiotherapy

## Abstract

**Purpose:**

We launched this prospective phase II single-arm trial on the combination of moderately hypo-fractionated radiotherapy and S-1, to explore the safety and efficacy of the new potent regimen in inoperable locally advanced esophageal squamous carcinoma (LA-ESCC) patients.

**Methods:**

Patients with unresectable stage II-IVB LA-ESCC (UICC 2002, IVB only with metastatic celiac or supraclavicular lymph nodes) were included. Moderately hypofractionated radiotherapy (60Gy in 24 fractions) concurrent with S-1 was delivered. Meanwhile, gastrostomy tube placement by percutaneous endoscopic gastrostomy (PEG) was performed to provide nutritional support. Nutritional supplements were prescribed to meet requirements. The study outcomes included objective response rate (ORR), progression-free survival (PFS), overall survival (OS), locoregional progression-free survival (LRPFS), distant metastasis-free survival (DMFS), failure pattern, toxicities, nutritional status and treatment compliance. Endoscopy was routinely performed during post-treatment follow-up.

**Results:**

Fifty-eight patients were included with a median follow-up of 24.4 months. The median age was 63 years (range 49-83 years) and 42 patients (72.4%) had stage III or IV diseases. The ORR was 91.3% and the CR rate was 60.3%. The estimated 2-year PFS rate and 2-year OS rate was 44.2% (95% confidence interval (CI), 31.3-57.1%) and 71.4% (95% CI, 59.4-83.4%), respectively. Radiation-induced esophagitis was the most common non-hematologic toxicity and 5 patients (8.6%) developed grade≥3 esophagitis. While, with PEG nutrition support, the nutrition-related indicators presented a clear trend toward a gradual improvement. Treatment-related death was not observed.

**Conclusions:**

The moderately hypo-fractionated radiotherapy combined with S-1 showed promising loco-regional disease control and survival benefit in inoperable LA-ESCC patients. Meanwhile, favorable nutritional status and low incidence of severe radiation-induced esophagitis were observed with PEG nutritional support. Moreover, endoscopy examination contributed to the early detection of recurrent esophageal lesions and timely salvage treatment. The efficacy and toxicity of the combined regimen deserved further evaluation.

**Trial registration:**

Clinicaltrials.gov, identifier NCT03660449.

## Introduction

Based on the results of a series of Radiation Therapy Oncology Group (RTOG) studies, concurrent chemoradiotherapy (CCRT) had been considered as the standard therapeutic strategy for inoperable locally advanced esophageal squamous cell carcinoma (LA-ESCC) patients (1–3). However, approximately 45-55% of LA-ESCC patients developed disease recurrences after definitive CCRT with locoregional relapse as the predominant treatment failure pattern (1, 2). To enhance the local control rate, some studies attempted to increase the fraction dose or total radiation dose to gross tumors. Nevertheless, the results of the landmark RTOG9405 trial showed that total dose escalation with conventional fraction failed to improve locoregional control (1). Another RTOG9012 trial found that escalating the radiation dose to 64.8 Gy did not confer a benefit compared with standard doses and may have contributed to a higher incidence of treatment-related severe toxicities (4). Recently, the ARTDECO study also approved that the total radiation dose escalation from 50.4 Gy to 61.6 Gy for esophageal carcinoma did not result in an improvement in local tumor control or survival (5). For that reason, fraction dose modification may be an option instead of total dose escalation. A trial conducted by Song demonstrated that moderately hypofractionated radiation could improve the local control rate with tolerable toxicities in inoperable LA-ESCC patients (6), which implied that moderately hypofractionated radiotherapy might offer therapeutic benefits with improved tumor control by increasing biologically effective doses (BED).

Although the concurrent dual-drug chemotherapeutic regimen was usually regarded as the preferred choice in previous studies (7, 8), it increased the incidence of serious adverse effects. In our previous phase I dose-escalation study of concurrent dual-drug chemotherapy, the results showed that the incidence of Grade 3-4 esophagitis and lymphopenia were 46.7% and 80%, respectively (9). Similarly, RTOG 8501 study reported that the rates of Grade 3-4 hematological toxicities and gastrointestinal reactions were 48% and 33%, which led to CCRT interruption and discontinuation (3). In a series of previous studies of the clinical outcomes of CCRT such as esophageal cancer, head and neck cancer, an interruption of treatment was found to be a negative factor in the local control (10, 11). McCloskey et al. reported that radiotherapy interruption longer than 1 week was a significant predictor of worse local control after definitive CCRT (12). Therefore, potent new CCRT regimens with lower toxicity appeared to be crucial for inoperable LA-ESCC patients for the improvement of treatment compliance and outcomes.

S-1, an oral fluoropyrimidine anticancer drug, is composed of tegafur (prodrug of fluorouracil), gimeracil (dihydropyrimidine dehydrogenase inhibitor) and oteracil potassium (13). After oral intake, tegafur is gradually converted to 5-FU to exert anticancer effects by inhibiting DNA synthesis by competitive inhibition of thymidylate synthetase and incorporation into RNA and DNA. While the gimeracil component could inhibit the degradation of 5-FU to maintain high plasma concentrations. And the oteracil selectively inhibits the formation of 5-FU nucleotides in the gut, thereby reducing gastrointestinal side effects (14). In previous studies, S-1 as concurrent chemotherapy agents in combination with radiotherapy yielded satisfactory survival outcomes with tolerable toxicities in older patients with esophageal carcinoma (15, 16). A recent phase III trial showed that there were no significant differences in the incidence of grade 3 or higher toxic effects between the S-1 CCRT and RT alone groups, except that grade 3 or higher leukopenia occurred in more patients in the CCRT group (9.5% *vs* 2.7%, P=0.01) (17). Notably, treatment-related deaths were observed in 3 patients (2.0%) in the CCRT group due to radiation-associated pneumonitis. In this context, intensive nutritional support is important for improving treatment tolerance and reducing toxicity in esophageal squamous cell carcinoma (ESCC) patients (18).

Therefore, we hypothesized that the moderately hypo-fractionated radiotherapy and concurrent S-1 would be able to reduce systemic toxicity and enhance loco-regional disease control; moreover, intensive percutaneous endoscopic gastrostomy (PEG) nutritional support and oral diet restriction during CCRT could be helpful for weight gain and reduce swallowing pain caused by radiation-induced esophagitis. We launched this prospective phase II clinical trial on the combination of moderately hypo-fractionated radiotherapy as well as concurrent S-1 and PEG nutritional support, to explore the safety and efficacy of the new potent regimen in inoperable ESCC patients.

## Materials and methods

### Ethics

The study protocol was reviewed and approved by the institutional review board. Each participant signed an informed consent form before commencing the trial. And the study was conducted in accordance with the requirement of the Declaration of Helsinki.

The study protocol was registered at ClinicalTrials.gov (NCT03660449) and was included in the **Supplementary Materials**.

### Inclusion and exclusion criteria

Study subjects were recruited between November 2017 and November 2019. All eligible patients met: 1) histologically confirmed ESCC; 2) II-IVB stages (IVB stage only with metastatic celiac or supraclavicular lymph nodes) based on the TNM staging system proposed by the International Union Against Cancer (UICC 2002) (19); 3) Eastern Cooperative Oncology Group (ECOG) performance status score of 0-1; 4) Charlson Comorbidity Index score ≤ 4; 5) capability of oral medication despite esophageal obstruction; 6) adequate physical condition to tolerate PEG. The main exclusion criteria included: 1) contraindication for radiotherapy or chemotherapy; 2) prior malignancies, except for curable non-melanoma skin cancer or cervical carcinoma in situ; 3) distant metastasis, except for celiac or supraclavicular lymph nodes metastases.

### Radiotherapy

Patients were fixed in vacuumed pad in the supine position with both arms straight beside the body. The gross tumor volume (GTV) contained the primary tumor and metastatic lymph nodes identified by pre-treatment work-ups. The clinical tumor volume (CTV) consists of the primary tumor plus a 3-cm craniocaudal margin and a 1-cm circumferential margin, and metastatic lymph nodes plus a 0.5- to 1-cm expansion margin. For lower-thoracic ESCC, the paracardial, lesser gastric curvature and the left gastric artery nodes were included in CTV, while for cervical and upper-thoracic ESCC, the supraclavicular area was included. The planning target volume (PTV) was derived from expanding GTV and CTV with a 0.5cm margin in all directions, respectively. A total dose of 60Gy with a fraction dose 2.5Gy was delivered to PTV-GTV and 40Gy in 16 fractions to PTV-CTV using a linear accelerator with 6- to 10-MV photons. And at least 95% of PTV volume received 95% of the prescription dose. The dose constraints to the organs at risk were as follows: Dmax of PTV-GTV ≤63Gy, V20 of lungs ≤ 30%, Dmean of lungs ≤13Gy, Dmax of spinal cord ≤45Gy, V30 of heart ≤30%, V30 of liver ≤20%, V5 of kidney ≤ 10%. In the study, intensity-modulated radiation therapy (IMRT) technology was applied for treatment planning and delivery. In addition, image guidance with cone-beam CT (CBCT) was performed weekly to verify the tumor position and ensure the precision of radiotherapy.

### Chemotherapy

S-1 was administered at 40mg/m2 twice daily within half an hour after meals on days 1-14 and 22-35 during treatment. In the event of Grade 2 thrombocytopenia, anemia, hepatic or renal dysfunction, Grade 3 leukopenia/neutropenia, Grade 2 radiation esophagitis, pneumonitis and other Grade 2 non-hematological toxicities, the dosage of S-1 was reduced by 25%. If more severe toxicity occurred S-1 was suspended. However, if the adverse events degraded to Grade 0-1 within 1 week of drug withdrawal, the patient could retake S-1 at 75% of the original dose, otherwise, S-1 was terminated henceforward.

### Percutaneous endoscopic gastrostomy and nutritional intervention

**Figure 1:**
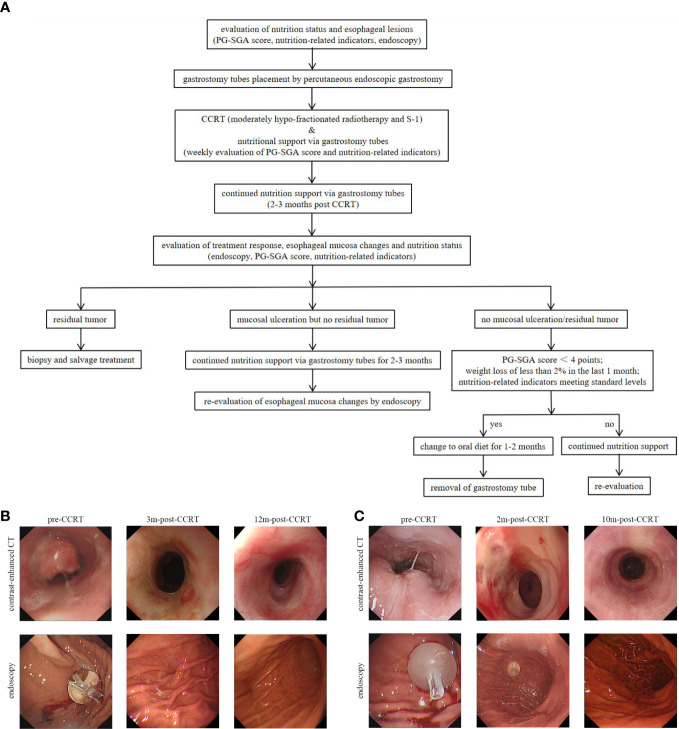
**(A)** The study protocol for percutaneous endoscopic gastrostomy and endoscopy evaluation. **(B)** A 76-year-old, male patient was diagonsed with middle thoracic ESCC. Three months after the completion of CCRT, the endoscopy showed no ulcer or tumor residue and the gastrostomy tube was removed. **(C)** A 62-year-old, male patient was diagnosed with upper thoracic ESCC. The patient got complete remission at 2 months after the completion of CCRT, but esophageal ulcers were found in the endoscopic examination. And the gastrostomy tube placement was prolonged to 10 months post-CCRT till the mucosa was completely repaired. *ESCC*, esophagus carcinoma; *CCRT*, concurrent chemoradiotherapy; *PG-SGA*, patient-generated subjective global assessment; *2m-post-CCRT*, 2 months after CCRT; *3m-post-CCRT*, 3 months after CCRT; *10m-post-CCRT*, 10 months after CCRT; *12m-post-CCRT*, 12 months after CCRT.

One week before CCRT, eligible patients received percutaneous endoscopic gastrostomy. Gastrostomy tubes were placed using the “pull” method introduced by Ponsky et al (20). In this method, a thin string was inserted into the stomach through a needle in the abdominal wall, grabbed with endoscopic biopsy forceps and then taken out through the esophagus and mouth. Subsequently, the cord was fixed to the outer end of the gastrostomy tube and the tube was pulled from the mouth to the esophagus, stomach, and out through the abdominal wall. Proton pump inhibitors and antibiotics were routinely used for 3 days after PEG. Subsequently, patients need to follow a strict oral diet protocol: 1)all nutritional supplements were given *via* gastrostomy tube during and 2-3 months post-CCRT; 2) Only saline solution, purified water and S-1 were allowed to be taken orally before gastrostomy tube removal; 3) oral liquid diet could be recovered when endoscopy showed no ulcer or residual tumor, patient-generated subjective global assessment (PG-SGA) score<4 points, weight loss less than 2% within 1 month and the nutrition-related indicators meeting standard levels (hemoglobin≥100g/L, albumin≥35g/L and prealbumin≥20mg/mL); 4) It was recommended to retain the gastrostomy tube for 1-2 months after resuming oral liquid diet, and then removed the tube if there are no abnormalities (**Figures 1A**, **1B**)

From the initiation of the treatment to 6 months post-CCRT, the PG-SGA score and nutrition-related indicators including weight, total protein, albumin, prealbumin, hemoglobin and lymphocyte were monitored and recorded regularly. Based on the PG-SGA score and the nutrition-related indicators, individualized and dynamic nutritional interventions were conducted on all subjects. All nutritional supplements were prescribed to meet final nutritional requirements (30-35 kcal/kg/d of energy, 30-40ml/kg/d of liquid and 1.2-1.5g/kg/d of protein).

### Toxicity

Radiation-induced esophagitis was evaluated weekly during treatment and four weeks after the completion of CCRT. Acute radiation pneumonitis was assessed from the initiation of treatment to 90 days after cessation of therapy. Other acute toxicities were recorded at baseline, weekly during CCRT and two weeks after the completion of therapy. All treatment-related toxicities were graded in terms of the Common Terminology Criteria for Adverse Events (CTCAE) of the National Cancer Institute (version 4.0). Serious adverse events (SAE) must be reported to the institutional review board within 24h and dealt with properly.

### Response evaluation and follow-up

At six to eight weeks after the completion of CCRT, treatment response was evaluated based on clinical work-ups including barium video-esophagography, contrast-enhanced computed tomography (CT) of neck, chest and abdomen, contrast-enhanced chest magnetic resonance imaging (MRI), upper gastrointestinal endoscopy and biopsy (if necessary). Positron emission tomography/computed tomography (PET/CT) scans were encouraged. And the Response Evaluation Criteria in Solid Tumors (RECIST) was applied to classify tumor response as progressive disease (PD), stable disease (SD), partial response (PR) or complete response (CR). All patients were then followed every 3 months for the first 2 years, every 6 months for years 3-5 and yearly thereafter. Every follow-up included history, physical examination, blood routine, basic metabolic panel, contrast-enhanced CT of the neck, thorax and abdomen, contrast-enhanced chest MRI, endoscopy and biopsy (if necessary). Notably, endoscopy was routinely performed at each post-treatment follow-up in the study.

### Statistical analysis

Descriptive statistics were conducted for the analysis of baseline characteristics and treatment-related toxicities. The objective response rate (ORR) was defined as the rate of complete response and partial response. Overall survival (OS) was calculated from treatment start to the date of death from any cause or censored at the last follow-up. Progression-free survival (PFS) was calculated from treatment start to the date of locoregional failure or distant metastasis or death, whichever occurred first. Locoregional progression-free survival (LRPFS) was calculated from treatment start to the date of the diagnosis of a locoregional recurrence as the first event. Distant metastasis-free survival (DMFS) was calculated from the treatment start to the date of distant metastasis. All data analyses were conducted using SPSS version 22.0. The survival rates were estimated using the Kaplan-Meier method and differences in the survival curves were compared by the log-rank test. Statistical comparisons were performed by using unpaired t-tests. P<0.05 was considered statistically significant.

## Results

### Patient characteristics

**Figure 2:**
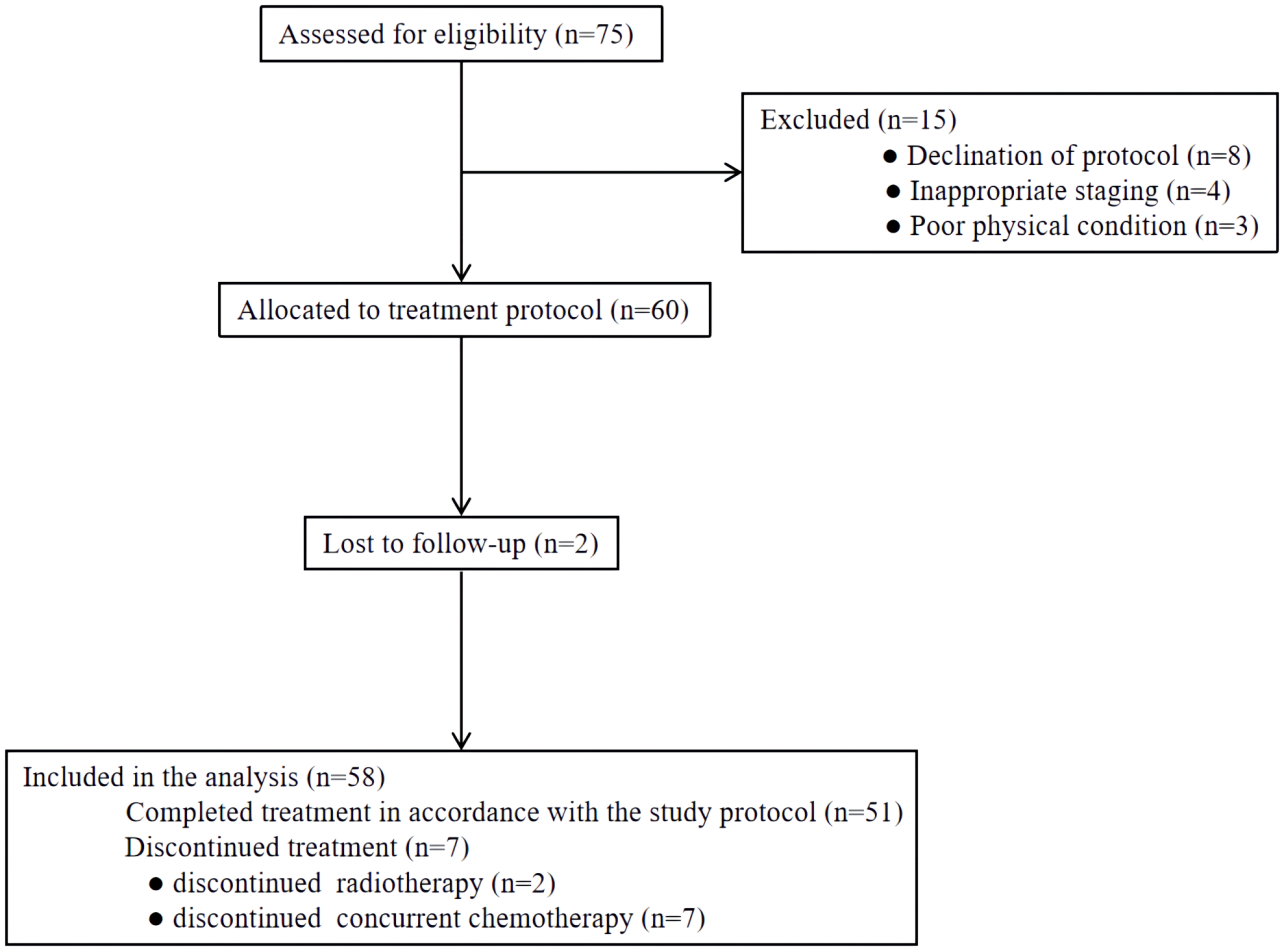
The flow chart of patient enrollment.

From November 2017 and November 2019, a total of 75 patients were screened at our medical institution. Seventeen patients were excluded for declining protocol-specific treatment or lost to follow-up. The remaining 58 patients were included in the final analysis (**Figure 2**). Overall, the median age of included patients was 63 years (range 49-83 years). Males comprised a majority of the cohort (75.9%), as did upper or middle thoracic tumors. Sixteen (27.6%) patients had stage IIB diseases and 35 (60.3%) patients had stage III diseases. Three patients with middle thoracic ESCC were classified as stage IVB for supraclavicular lymph node metastases. Detailed characteristics are presented in **Table 1**.

**Table 1:** Patient characteristics for the whole cohort (n=58).

Characteristics	No.	%
Age(years)		
median	63	
range	49-83	
Sex		
female	14	24.1%
Male	44	75.9%
ECOG PS		
0	34	58.6%
1	24	41.4%
Location of primary tumor		
cervical esophagus	8	13.8%
upper thoracic esophagus	20	34.5%
middle thoracic esophagus	22	37.9%
lower thoracic esophagus	6	10.4%
synchronous multiple primary cancer	2	3.4%
cT stage		
T2	17	29.3%
T3	31	53.5%
T4	10	17.2%
cTNM Stage		
IIB	16	27.6%
III	35	60.3%
IVA	4	6.9%
IVB	3	5.2%

### Treatment details and responses

Fifty-six patients completed radiotherapy as per protocol, while two patients only completed 19 of 24 fractions (47.5Gy) because of persistent Grade 3 esophagitis and Grade 3 leukopenia, respectively. Radiation therapy interruption occurred in six patients (10.4%). Seven patients discontinued concurrent chemotherapy due to gastrointestinal (n=4) and hematological toxicities (n=3). Chemotherapy dose reduction occurred in 12.1% of patients. Notably, all patients received PEG before treatment for nutritional intervention.

Six to eight weeks after the completion of CCRT, 35 (60.3%) patients were assessed as CR and 18 (31.0%) patients as PR. The ORR was 91.3%.

### Survival and failure patterns

**Figure 3:**
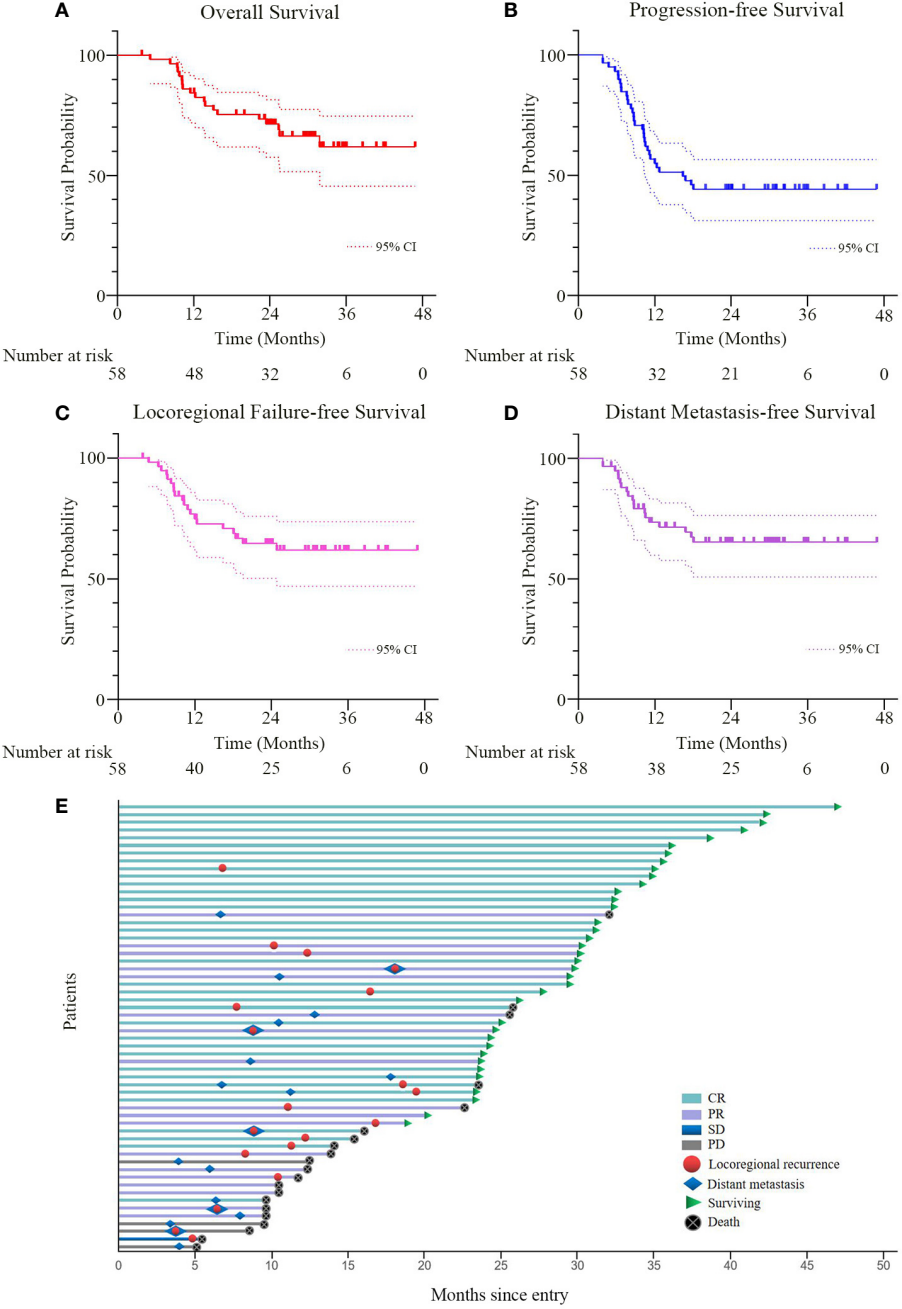
**(A–D)** Overall survival, progression-free survival, locoregional progression-free survival, and distant metastasis-free survival curves for all patients. The broken lines represent the upper and lower limits of the 95% CI of the survival curve, respectively. **(E)** Tumor swimmer plot for all patients. CR, PR, SD and PD were evaluated by the RECIST criteria based on CT, MRI, endoscopy and biopsy at six to eight weeks after the completion of CCRT. *CCRT*, concurrent chemoradiotherapy; *RECIST*, response evaluation criteria in solid tumors; *CR*, complete remission; *PR*, partial remission; *SD*, stable disease; *PD*, progressive disease.

The median follow-up duration was 24.4 (range 3.9-46.8) months and the estimated 2-year OS rate was 71.4% (95% confidence interval (CI), 59.4-83.4%, **Figure 3A**). During the follow-up period, 12 patients (20.7%) had locoregional recurrence as the first failure. Distant metastases as the first failure occurred in 14 (24.1%) patients (lung, n=5; out-of-field nodal, n=5; abdomen, n=2; multiple organs metastases, n=2). Moreover, 5 (8.6%) patients had both locoregional recurrence and distant metastases as the first failure (**Figure 3E**). The estimated 2-year PFS rate was 44.2% (95%CI, 31.3-57.1%, **Figure 3B**) and the median PFS was 16.4 (95%CI, 9.3-23.6) months. The estimated 2-year LRPFS rate and DMFS rate were 64.6% (95%CI, 51.7-77.5%, **Figure 3C**) and 65.1% (95%CI, 52.4-77.8%, **Figure 3D**), respectively. Patients with progressive disease received timely salvage therapy, with 12 patients receiving systemic chemotherapy alone, 9 patients receiving a combination of immunotherapy and chemotherapy, and 5 patients receiving re-radiation combined with chemotherapy.

### Toxicity

All acute toxicities are reported in **Table 2**. The most frequent non-hematologic toxicity was esophagitis. 36 patients (62.1%) had grade 2 esophagitis and 5 patients (8.6%) developed grade≥3 esophagitis. Radiation-induced esophagitis is mainly characterized by esophagus pain, and strong opioids were administered to 8 patients for pain relief. Only one patient occurred with esophago-mediastinal fistula and none presented with grade 3-4 pneumonitis in the study. The most common grade≥3 hematological toxicity was lymphopenia (22/58, 37.9%), followed by leukopenia (2/58, 3.4%).

**Table 2:** Treatment-related adverse events (n=58).

Adverse event	Grade, n (%)			
	1	2	3	4
Hematological				
anemia	15(25.9%)	7(12.1%)	0	0
leukopenia	23(39.7%)	13(22.4%)	2(3.4%)	0
lymphopenia	5(8.6%)	30(51.7%)	22(37.9%)	0
neutropenia	7(12.1%)	4(6.9%)	0	0
thrombocytopenia	10(17.2%)	1(1.7%)	0	0
Liver				
hyperbilirubinemia	9(15.5%)	0	0	0
increased liver enzyme	18(31.0%)	0	0	0
Gastrointestinal				
esophagitis	16(27.6%)	36(62.1%)	5(8.6%)	0
esophageal fistula	0	1(1.7%)	0	0
nausea	18(31.0%)	9(15.5%)	1(1.7%)	0
vomiting	9(15.5%)	9(15.5%)	1(1.7%)	0
anorexia	27(46.6%)	9(15.5%)	1(1.7%)	0
diarrhea	6(10.4%)	6(10.4%)	2(3.4%)	0
constipation	4(6.9%)	0	0	0
Pulmonary				
pneumonitis	33(56.9%)	8(13.8%)	0	0
cough	14(24.1%)	19(32.8%)	0	0
dyspnea	1(1.7%)	0	0	0
chest pain	2(3.4%)	0	0	0
hoarseness	4(6.9%)	0	0	0
General				
dermatitis	16(27.6%)	5(8.6%)	1(1.7%)	0
fatigue	29(50.0%)	6(10.4%)	0	0

### Longitudinal changes of nutritional status

**Figure 4:**
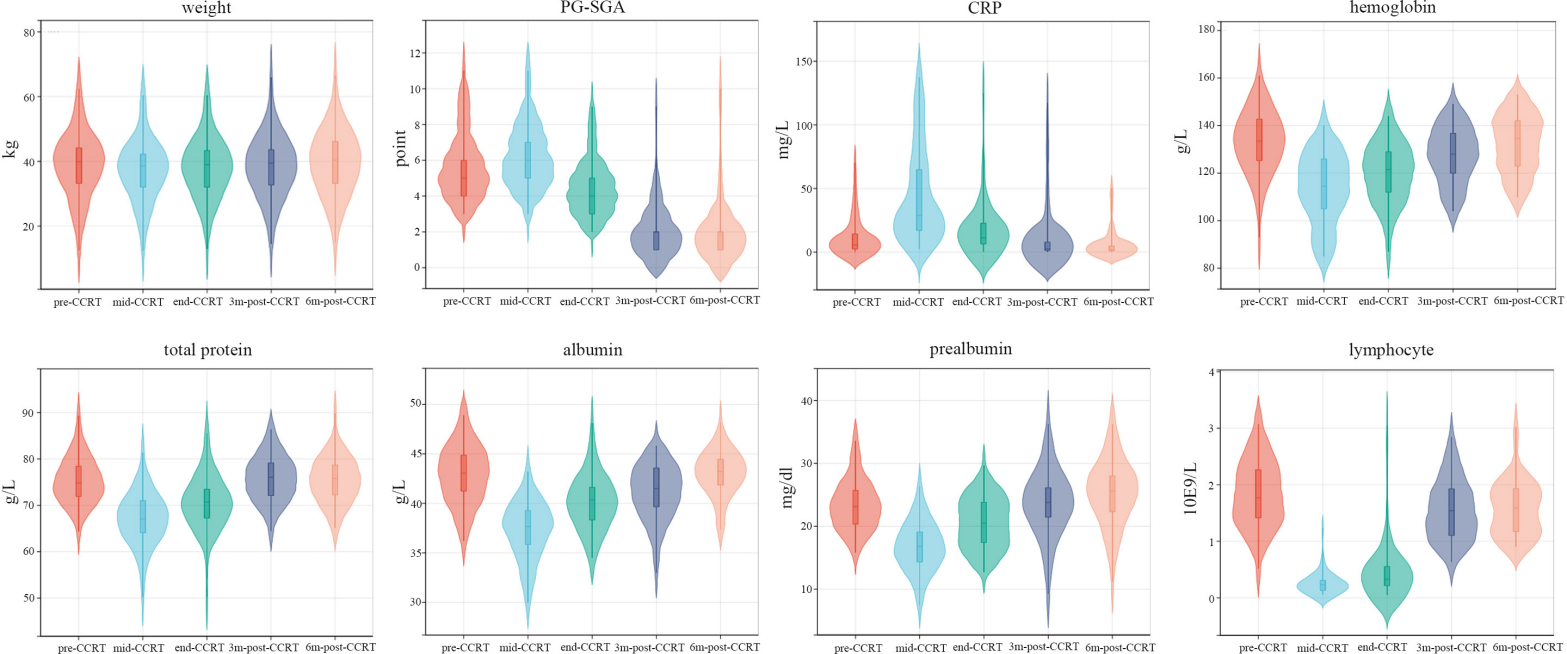
The changes longitudinal of nutrition-related indicators. *mid-CCRT*, 2 weeks after the start of CCRT; *end-CCRT*, the end of CCRT; *3m-post-CCRT*, 3 months after CCRT; *6m-post-CCRT*, 6 months after CCRT.

The changes of nutrition-related indicators are presented in **Figure 4**. As illustrated, the nutrition-related indicators including hemoglobin, total protein, albumin, prealbumin and lymphocyte significantly decreased in the early stage of treatment. However, with intensive nutrition intervention, the nutrition-related indicators presented a clear trend toward a gradual improvement and returned to initial levels within 3 to 6 months post-CCRT. Meanwhile, the body weight of patients remained stable throughout the treatment. 47 patients (81.0%) removed the gastrostomy feeding tube at the first or second follow-up, while 3 patients (5.2%) retained the tube for≥12 months. The median duration of gastrostomy tube retention was 4.1 (range 2.9-12.5) months.

### Endoscopy evaluation and follow-up

**Figure 5:**
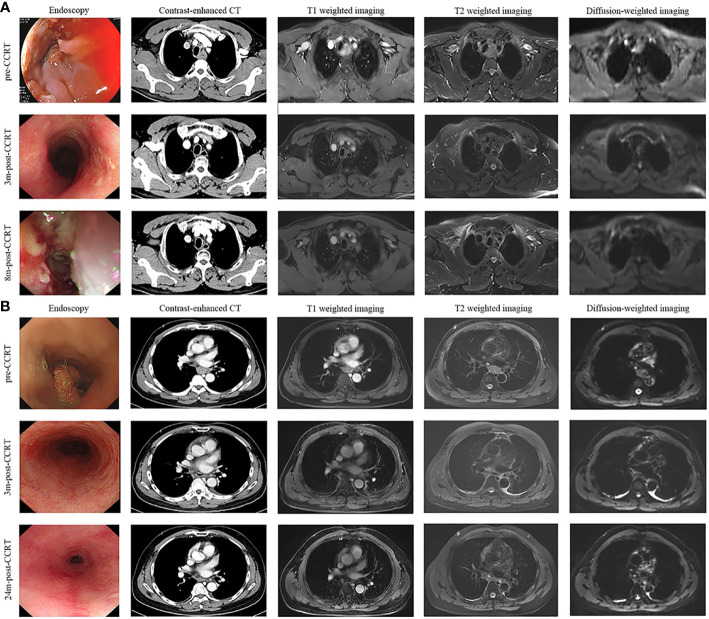
Disease evaluation by endoscopy, CT, T1-weighted, T2-weighted and diffusion-weighted sequences of MRI. **(A)** A 56-year-old, male patient was diagnosed with ESCC, which was located in the upper and middle thoracic esophagus. The treatment response of the case was assessed as CR after receiving CCRT. At 8 months after the completion of treatment, imaging examination showed no obvious abnormalities, but endoscopic examination revealed a small ulcerative lesion (red arrow) and pathological biopsy confirmed recurrence. **(B)** A 67-year-old, male patient was diagnosed with ESCC, which is located in the middle thoracic esophagus. The patient achieved CR after receiving CCRT and remained disease-free status in the 2-year follow-up period. *ESCC*, esophagus carcinoma; *CCRT*, concurrent chemoradiotherapy; *CR*, complete remission; *3m-post-CCRT*, 3 months after CCRT; *8m-post-CCRT*, 6 months after CCRT; *24m-post-CCRT*, 24 months after CCRT.

Notably, endoscopy was routinely performed at each post-treatment review in the study. Compared with imaging examination alone, endoscopy showed some obvious advantages: first, endoscopy could directly observe the changes in esophageal mucosa and help decide the time for extubation. As shown in **Figure 1C**, the patient got complete remission as assessed by imaging examination at two months after the completion of CCRT, but esophageal ulcers were found in endoscopic examination and no residual tumor was found in the pathological biopsy. In this case, the gastrostomy tube placement was prolonged until the mucosa was completely repaired; second, endoscopy contributed to the early detection of mucosal lesions. As presented in **Figure 5A**, the treatment response of the case was assessed as CR. At the third review after treatment, imaging examination showed no disease progression, but endoscopic examination revealed a small ulcerative lesion and pathological biopsy confirmed recurrence. Thus, the comprehensive examination method adopted in the study was more conducive to early detection of disease progression and timely subsequent treatment to prolong the overall survival time.

## Discussion

With regard to the combined efficacy of moderately hypo-fractionated radiotherapy, concurrent S-1 and PEG nutritional support, 35 of 58 patients (60.3%) got CR and the overall ORR was 91.3%. Meanwhile, the estimated 2-year OS rate and 2-year PFS rate were 71.4% and 44.2%, respectively. The gross tumor volume was reduced by 100% after the completion of CCRT in 60.3% of patients (35/58) with a pretreatment volume ranging from 21.9 to 203.3cm3. In addition, PEG nutritional support during CCRT was helpful to maintain the nutritional status of all subjects. Therefore, with the potent new CCRT regimens and nutritional management, promising tumor regression was achieved with moderated toxicities.

In clinical practice, CCRT has been broadly applied as the standard therapeutic strategy for LA-ESCC patients. But how to improve locoregional control and minimize treatment-related toxicities remains a tricky question. Recently, a phase I/II dose-escalation study conducted by Ma et al. indicated that a daily dose of ≤5Gy was safe in hypofractionated radiation for the treatment of ESCC (21). Meanwhile, compared to conventional radiation, moderately hypofractionated radiation could significantly decrease the risk of locoregional failure with a clear tendency toward additional survival benefits (6, 21). Therefore, moderately hypofractionated radiation with a fraction dose of 2.5Gy was adopted in the study. In the proposed chemotherapy regimen, we replaced the classical dual-drug regimen (capecitabine/fluorouracil&platinum) with S-1, a single chemotherapeutic drug taken orally. Compared with continuous infusion of fluorouracil, S-1 provides a more convenient way of administration and a superior radiosensitizing effect (13, 14, 22–24).

Our study achieved a promising treatment response by combing moderately hypofractionated radiation with a single chemotherapeutic agent. Assessing treatment response at six to eight weeks after the completion of CCRT, we found that 35 (60.3%) patients achieved CR and the ORR was 91.3%. In the majority of published clinical trials where patients received dual-drug regimen chemotherapy with concurrent radiotherapy, the reported ORR was approximately 75-90% (25–27). Thus, compared with the previous data, our study presented a satisfactory tumor treatment response. After close long-term follow-up, 12 patients (20.7%) were found to have the locoregional recurrence as the first failure by endoscopy examination. Distant metastases as the first failure occurred in fourteen (24.1%) cases. Another 5 (8.6%) patients presented both locoregional disease and distant metastases as the first failure. Compared with the data reported by RTOG 8501 and RTOG 9405 (locoregional failure rate was as high as 50% and 56%, respectively) (1, 3), the incidence of locoregional recurrence in the current study was relatively lower.

As reported by previous RTOG trials, severe acute esophageal toxicities (grade≥3) occurred in 25-60% of ESCC patients who received chemoradiation (1–4). In our study, the incidence of grade 3 esophagitis and adverse gastrointestinal reactions was 8.6% and 5.1%, respectively. Only one patient developed esophago-mediastinal fistula and none presented with grade 3-4 pneumonitis. These results demonstrated that PEG nutritional support and oral diet restriction were helpful to reduce esophageal friction injury and promote repair, thereby reducing acute esophageal toxicities. Meanwhile, intensive nutritional interventions also exerted a positive effect in maintaining nutritional status, reducing adverse events and improving the immune function of patients with esophageal carcinoma. Overall, the new regimen recommended by our study showed a safe toxicity profile.

As is well-known, the incidence of malnutrition in esophageal carcinoma patients ranks first among all types of malignancies, reaching 67% to 85% (28, 29). Relevant literature reported that malnutrition not only reduces the sensitivity of chemoradiotherapy but also increases treatment-related toxicities (30–32). As illustrated in **Figure 3**, the nutrition-related indicators significantly decreased in the early stage of treatment. However, with active nutrition intervention, the nutrition-related indicators presented a clear trend toward a gradual improvement and returned to initial levels within 3 to 6 months post-CCRT. Meanwhile, the body weight of patients remained stable throughout the treatment. Furthermore, the radiation-induced esophagitis was significantly reduced by intensive PEG nutritional support and oral diet restriction. Therefore, nutritional management during chemoradiotherapy is helpful to normalize food intake and improve the nutritional status of ESCC patients.

Recently, a multicenter randomized phase 3 clinical trial conducted by Chen et al. evaluated the efficacy and safety of CCRT with S-1 *vs* radiotherapy alone in older ESCC patients (17). In the CCRT group, the median PFS intervals were 18.7 (95% CI, 12.1-25.2) months and the 2-year OS rate was 53.2%. Although the median PFS interval was slightly shorter in our study, our protocol achieved a significantly longer 2-year OS rate of 71.4%. The possible reasons for the difference included: 1) the age composition of our study is relatively young with a median age of 63 years versus 77 years in Chen’s study; 2) the hypofractionated radiation technique helped to improve the locoregional control; (**Figure 5B**). 3) endoscopy was conducted as the main examination method at each post-treatment review, which allowed early detection of recurrent disease and timely salvage treatment.

Several limitations of the current study should be taken into consideration. Firstly, this was a single-center prospective study with a relatively small sample size which may limit the generalization of our results. Secondly, although a total radiation dose of 50.4 Gy with a conventional fraction is recommended by the guidelines of National Comprehensive Cancer Network (NCCN), we delivered a total dose of 60Gy in the study. Considering ESCC as the major histological type of esophageal cancer in China, it is more susceptible to locoregional recurrence than esophagus adenocarcinoma (33, 34). Recently, the randomized ARTDECO study approved that radiation escalation from 50.4Gy to 61.6Gy for esophageal tumors did not result in an improvement in local tumor control or survival and a dose of 50.4Gy could yield acceptable locoregional and survival outcomes (5). But in the ESCC subgroup, the 3-year local progression-free survival rate and LRPFS rate were both numerically better in the high dose group than in the standard group, although the difference was not statistically significant. Similar results were confirmed by a phase III multicenter randomized study of the radiation dose of 60Gy *vs* 50Gy in CCRT for inoperable ESCC (35). Meanwhile, the study reported that the difference in the incidence of ≥grade 3 all adverse events in the two groups was not statistically significant (P=0.5). Moreover, a total dose beyond 60Gy was also supported by some published literature as well as the guidelines of Chinese Society of Clinical Oncology (36–39). Thus, the optimal total radiation dose for ESCC remains controversial and the radiation dose adjustment remains to be a focus of future clinical studies.

## Conclusions

Although several limitations exist, our study showed that the moderately hypo-fractionated radiotherapy combined with S-1 showed promising loco-regional disease control and survival benefit in inoperable LA-ESCC patients. Meanwhile, favorable nutritional status and low incidence of severe radiation-induced esophagitis were observed in subjects receiving PEG nutritional support. Moreover, endoscopy examination contributes to the early detection of recurrent esophageal lesions and timely salvage treatment. The efficacy and toxicity of the combined regimen deserved further evaluation.

## Data availability statement

The raw data supporting the conclusions of this article will be made available by the authors, without undue reservation.

## Ethics statement

The studies involving human participants were reviewed and approved by Sun Yat-sen University Cancer Center. The patients/participants provided their written informed consent to participate in this study. Written informed consent was obtained from the individual(s) for the publication of any potentially identifiable images or data included in this article. 

## Author contributions

Study conception and design: RZ, GL, SG, BQ and HL. Literature review: RZ and BQ. Data acquisition: YW, QL, DW, NC, FL, JG, and WY. Statistical analysis: RZ, FL, DW, NC, and BQ. Data interpretation: RZ, GL, SG, BQ and HL. Manuscript preparation: RZ, GL, SG, and HL. Manuscript review: All authors. All authors contributed to the article and approved the submitted version.
