# Supplementary material for: Moderately hypo-fractionated radiotherapy combined with S-1 in inoperable locally advanced esophageal squamous cell carcinoma: A prospective, single-arm phase II study (GASTO-1045)
Source: Front Oncol. 2023 Mar 10;13:1138304. doi: 10.3389/fonc.2023.1138304 (PMC10036360; doi:10.3389/fonc.2023.1138304)
Supplement: Supplementary file 1 [file Presentation_1.pdf]

Moderately Hypo-fractionated Radiotherapy Combined with S-1 in Inoperable Locally Advanced Esophageal Squamous Cell Carcinoma: A Prospective, single arm Phase II Study

Document type: Clinical Trial Protocol

Development phase: II

Table of contents

Table of contents ... 2
List of abbreviations ... 4
Protocol synopsis ... 6
1 Introduction ... 9
1.1 Background ... 9
2 Study objectives ... 13
2.1 Primary endpoint ... 13
2.2 Secondary endpoints ... 14
3 Investigational plan ... 14
3.1 Study design ... 14
3.2 Research progress and completion time ... 15
4 Population ... 15
4.1 Sample size ... 15
4.2 Inclusion criteria ... 15
4.3 Exclusion criteria ... 16
4.4 Premature patient withdrawal ... 16
5 Treatment ... 17
5.1 Schedule of study ... 17
5.2 Concurrent chemoradiotherapy ... 17
5.2.1 Concurrent chemotherapy ... 17
5.2.2 Radiotherapy ... 17
5.3 Percutaneous endoscopic gastrostomy (PEG) ... 21
6 Adverse events and treatment plan adjustment ... 22
6.1 Definition ... 22
6.1.1 Adverse events (AE) ... 22
6.1.2 Serious adverse events (SAE) ... 23
6.2 Toxicity of radiotherapy and management of radiation-related side effects ... 23
6.3 Toxicity of chemotherapy and adjustment of chemotherapy regimen ... 24
6.3.1 Common adverse events of chemotherapy ... 24
6.3.2 Chemotherapy dose adjustment ... 25
7 Combined medication ... 25
7.1 Prophylactic medication ... 25
7.2 Concomitant medication ... 25
7.2.1 Supportive care ... 25
7.2.2 Other drugs ... 25
8 Visit schedule and assessments ... 25
8.1 The baseline assessment ... 26
8.2 The assessment during the treatment ... 26
8.3 The efficacy assessment at two months after the completion of treatment ... 27
8.4 Quality of life score ... 27
8.5 Follow up ... 28
9 Evaluation of the research results ... 28
9.1 Efficacy ... 28
9.1.1 Evaluation criteria for the treatment response ... 28
9.1.2 Objective response rate ... 28
9.1.3 Survival outcomes ... 29
9.1.4 Nutritional status ... 29
9.1.5 Failure patterns ... 29
9.2 Safety assessment ... 29
9.2.1 Overall safety ... 29
9.2.2 Clinical safety ... 29
9.2.3 Laboratory evaluations ... 30
9.2.4 Appropriateness of safety measurements ... 30
10 Statistics ... 30

10.1 Analysis sets 30
10.2 Patient demographics and other baseline characteristics 31
10.3 Analysis of the primary and key secondary variables 31
10.3.1 Variables 31
10.3.2 Statistical model and method of analysis 31
11 Ethical considerations 31
11.1 Regulatory and ethical compliance 31
11.2 Informed consent procedures 31
11.3 Responsibilities of the investigator and IRB/IEC 32
11.4 Publication of study protocol and results 32
12 Protocol adherence 32
12.1 Protocol amendments 33
13 Reference 33

List of abbreviations

AE	Adverse Event
ALT	ALanine aminoTransferase
AST	ASpartate aminoTransferase
BED	Biologically Effective Doses
BMI	Body Mass Index
CCRT	Concurrent Chemoradiotherapy
CR	Complete Response
CRF	Case Report/Record Form (paper or electronic)
CRO	Contract Research Organization
CTCAE	Common Terminology Criteria for Adverse Events
CTV	Clinical Target Volume
DVH	Dose-volume Histogram
ECOG	Eastern Cooperative Oncology Group
GCP	Good Clinical Practice
GTV	Gross Target Volume Dose-volume histogram
LA-ESCC	Locally Advanced Esophageal Squamous Cell Carcinoma
MIP	Maximum Intensity Projection
ICH	International Conference on Harmonization of Technical Requirements for Registration of Pharmaceuticals for Human Use
IEC	Independent Ethics Committee
ITV	Internal Target Volume
IMRT	Intensity-Modulated Radiotherapy
IN	Investigator Notification
IRB	Institutional Review Board
ORR	Objective Response Rate
OS	Overall Survival
PD	Progressive Disease
PEG	Percutaneous Endoscopic Gastrostomy
PFS	Progression-free Survival
PG-SGA	Patient-generated subjective global assessment
PR	Partial Response
PTV	Planned Target Volume
RECIST	Response Evaluation Criteria in Solid Tumors
RTOG	Radiation Therapy Oncology Group
SAE	Serious Adverse Event
SD	Stable Disease
SIB	Simultaneous Integrated Boost

SMART	Simultaneous Modulated Accelerated Radiotherapy
ULN	Upper Limit of Normal
WHO	World Health Organisation
WBC	White Blood Cell count

Protocol synopsis

Full title	Moderately Hypo-fractionated Radiotherapy Combined with S-1 in Inoperable Locally Advanced Esophageal Squamous Cell Carcinoma: A Prospective, single arm Phase II Study
Background	Based on the results of a series of Radiation Therapy Oncology Group (RTOG) studies, concurrent chemoradiotherapy (CCRT) had been considered as the standard therapeutic strategy for inoperable locally advanced esophageal squamous cell carcinoma (LA-ESCC) patients. However, approximately 45-55% of LA-ESCC patients developed disease recurrences after definitive CCRT with locoregional relapse as the predominant treatment failure pattern. Thus, how to improve the local control rate and reduce the recurrence rate has become the key to improve the efficacy of radiotherapy for esophageal cancer patients.<br>With the innovation of radiotherapy technology, intensity-modulated radiotherapy (IMRT) could improve the conformal degree of the target area, reduce the dose of organs at risk, and reduce the acute and late toxicity of organs. Moreover, studies have demonstrated that moderately hypofractionated radiotherapy might offer therapeutic benefits with improved tumor control by increasing biologically effective doses (BED). S-1 is a novel, orally administered fluorouracil that is a combination of tegafur, gimeracil and oterasil potassium. Compared with continuous infusion of fluorouracil, S-1 provides a more convenient way of administration and superior radiosensitizing effect, which has become a favorable factor in the treatment of patients with esophageal cancer.
Objectives	The primary objective is to assess the safety and efficacy of the combination of moderately hypo-fractionated radiotherapy as well as concurrent S-1 in inoperable LA-ESCC patients.<br>Primary endpoint:<br>progression-free survival (PFS)<br>Secondary endpoints:<br>objective response rate (ORR)、overall survival (OS), failure pattern, toxicities, nutritional status and treatment compliance
Study type	Interventional
Study design	Prospective, single-arm Phase II Study
Study Population	The study population will include:<br>• Males and females aged ≥18 years.<br>• LA-ESCC patients diagnosed with II-IVB stages (IVB stage only with metastatic celiac or supraclavicular lymph nodes) based on the TNM staging system proposed by the International Union Against Cancer (UICC 2002)
Sample Population	The Clopper-Pearson method was used to estimate the sample size. $\alpha=0.05$, $1-\beta=0.80$. The expected clinical response rate of patients with ESCC treated with radiotherapy concurrent with S-1 was 80%. Compared with the previous clinical response rate of 60%, A total of 35 subjects were required. Considering the elimination and shedding of cases during the trial, the number of cases was increased by 20% and the total of cases was determined to be ≥ 42.

Key inclusion criteria	• Histologically confirmed ESCC.<br>• II-IVB stages (IVB stage only with metastatic celiac or supraclavicular lymph nodes) based on the TNM staging system proposed by the International Union Against Cancer (UICC 2002)<br>• Eastern Cooperative Oncology Group (ECOG) performance status score of 0-1<br>• Charlson Comorbidity Index score≤4<br>• Capability of oral medication despite esophageal obstruction<br>• Adequate bone marrow, hepatic and renal function: White blood cell count ≥ 4×10^9/L, absolute neutrophil count ≥ 1.5×10^9/l, platelets ≥ 100×10^9/L, hemoglobin haemoglobin ≥ 100g/L, serum creatinine and bilirubin <1.5×upper limit of normal (ULN), transaminase <2 × ULN
Key exclusion criteria	• Contraindication for radiotherapy or chemotherapy<br>• Prior malignancies, except for curable non-melanoma skin cancer or cervical carcinoma in situ<br>• Distant metastasis, except for celiac or supraclavicular lymph nodes metastases<br>• Malignant pleual effusion or pericardial effusion<br>• Patients participating in any other clinical trial within 30 days before inclusion or concurrent with this study<br>• Uncontrollable seizures of loss of self-control due to mental illness<br>• Serious drug allergy history or allergic constitution
Study treatment	Concurrent chemoradiotherapy<br>• Radiotherapy: All patients receive IMRT, and the scheduled doses are given once per day, 5 days per week. A total dose of 60Gy with a fraction dose 2.5Gy was delivered to PTV-GTV and 40Gy in 16 fractions to PTV-CTV. And at least 95% of PTV volume received 95% of the prescription dose.<br>• Chemotherapy: S-1 was administered at 40mg/m2 twice daily within half an hour after meals on days 1-14 and 22-35 during treatment.
Key efficacy assessments	• PFS: PFS was calculated from treatment start to the date of locoregional failure or distant metastasis or death, whichever occurred first.<br>• OS: OS was calculated from treatment start to the date of death from any cause or censored at the last follow-up<br>• Treatment response: the Response Evaluation Criteria in Solid Tumors (RECIST) was applied to classify tumor response as progressive disease (PD), stable disease (SD), partial response (PR) or complete response (CR)<br>• ORR: ORR was defined as the rate of CR and PR<br>• Nutritional status: the patient-generated subjective global assessment (PG-SGA) score and nutrition-related indicators including weight, total protein, albumin, prealbumin, hemoglobin and lymphocyte need to be monitored and recorded regularly from the initiation of the treatment to 6 months post-CCRT.
Key safety assessments	• Adverse event monitoring<br>• Laboratory examinations of blood and urine

Statistics	Descriptive statistics were conducted for the analysis of baseline characteristics and treatment-related toxicities. All data analyses were conducted using SPSS version 22.0. The survival rates were estimated using the Kaplan-Meier method and differences in the survival curves were compared by the log-rank test. Statistical comparisons were performed by using unpaired t-tests. $P < 0.05$ was considered statistically significant.

1 Introduction

1.1 Background

1.1.1 Current treatment of locally advanced esophageal squamous cell carcinoma

Esophageal cancer is a common malignant tumor of the digestive tract, ranking 8th in the incidence rate and 6th in the mortality rate. China is a country with a high incidence of esophageal cancer. There are more than 400,000 new cases of esophageal cancer in the world every year, about 250,000 of which occur in China [1]. Among them, esophageal squamous cell carcinoma (ESCC) is the most common pathological type. Due to the lack of specificity symptoms, most patients with ESCC were found in the middle or late stages, and only 20% can be treated with radical resection. Based on the results of the Radiation Therapy Oncology Group (RTOG) 8501 trial, concurrent chemoradiotherapy (CCRT) has become the standard treatment for patients with locally advanced esophageal cancer who are inoperable or unwilling to undergo surgery. In the RTOG8501 trial, 123 patients were randomly assigned to either CCRT or radiotherapy alone. In the CCRT group, all patients received a dose of 50Gy/25F radiotherapy combined with DDP+5-FU chemotherapy every three weeks. While the dose of radiotherapy alone was 64Gy/32F. The results showed that the median overall survival (OS) and the 2-year survival rate were significantly improved in the CCRT group compared with radiotherapy alone. After long-term follow-up, the 5-year survival rate of patients with locally advanced esophageal squamous cell carcinoma (LA-ESCC) reached 26% [2].

At present, the radiotherapy regimen for esophageal cancer remains controversial. In the RTOG8501 trial, a radiotherapy dose of 30Gy/15F was given to the target area from the supraclavicular fossa to the esophagogastric junction. Then, the intensive radiotherapy with a dose of 20Gy/10F was delivered to the shrinking field including the tumor area plus a 5-cm craniocaudal margin. Moreover, the RTOG9405 study attempted to increase the total radiation dose to gross tumors. However, due to the large irradiation range and chemotherapy dose, the high-dose group (64.8Gy) showed no advantage over the 54Gy group in terms of survival and local control rate. Only 67% of patients in the high-dose group completed the treatment due to the high incidence of side effects [3]. Therefore, the current radiotherapy dose recommended

by the National Comprehensive Cancer Network (NCCN) guidelines is still 50.4Gy. In China, the concurrent chemotherapy dose and the radiotherapy range are relatively lower than the regimen recommended by the RTOG8501 trial. And the efficacy and side effects of CCRT with a dose of 60Gy in clinical practice are acceptable. Thus, the radiotherapy dose of 60Gy/30F is recommended by the guidelines of Chinese Society of Clinical Oncology.

However, approximately 45-55% of LA-ESCC patients developed disease recurrences after definitive CCRT with locoregional relapse as the predominant treatment failure pattern. Thus, a new treatment regimen needs to be explored.

1.1.2 The innovation of radiotherapy technology could increase the fraction dose and reduce the toxicity

The main reason for treatment failure of esophageal cancer patients who received CCRT is locoregional relapse [3, 4]. Most scholars believe that the improvement of the local control rate of radiotherapy for esophageal cancer will be transformed into an improvement in survival to a large extent. How to improve the local control rate of tumor and reduce recurrence rate has become the key to improving the efficacy of radiotherapy for esophageal cancer. The results of the RTOG9405 study showed that high-dose radiotherapy did not succeed in improving local control or survival. However, the radiotherapy technique used in the study was a two-dimensional conventional technique (2D-CRT). Moreover, large field irradiation and local tumor area augmentation result in a high dose of radiation to normal tissues, thereby increasing toxic and side effects, and masking the potential survival benefit of a high dose. How to protect normal tissues and reduce toxicity is the key to treatment. The main reason for treatment failure of esophageal cancer patients who received CCRT is locoregional relapse [3, 4].

Most scholars believe that the improvement of the local control rate of radiotherapy for esophageal cancer will be transformed into an improvement in survival to a large extent. How to improve the local control rate of tumors and reduce recurrence rate has become the key to improving the efficacy of radiotherapy for esophageal cancer. The results of the RTOG9405 study showed that high-dose radiotherapy did not succeed in improving local control or survival. However, the radiotherapy technique used in the study was a two-dimensional conventional technique (2D-CRT). Moreover, large field irradiation and local tumor area augmentation result in a high dose of radiation to normal tissues, thereby increasing toxic and

side effects, and masking the potential survival benefit of a high dose. How to protect normal tissues and reduce toxicity is the key to treatment.

Simultaneous modulated accelerated radiotherapy (SMART) technology allows simultaneous integrated boost (SIB) at the tumor site in the design of a radiotherapy plan, which indicates a high dose is given to the primary tumor site while a low dose is given to the subclinical tumor site or its surrounding prevention site. SIB reduces accelerated reproliferation of tumor clonogenic cells and exhibits better tumor control by increasing the dose of radiation at the tumor site and shortening the total radiation time [5-7]. Since the most local failures of ESCC patients who received CCRT occurred at the site of the primary tumor [8], we speculated that SIB technology could selectively increase the dose in areas with high recurrence risk. A MD Anderson study designed four radiotherapy plans for 10 patients with distal esophageal cancer: 2DCRT with a dose of 50.4Gy, 2DCRT with a dose of 64.8Gy, IMRT with a dose of 50.4Gy and SIB-IMRT with a dose of 64.8Gy [9]. All four radiotherapy plans adopted 28 fractions. Dosimetry showed that the mean irradiation dose of heart, lung and liver was significantly lower in the IMRT technology group with a total dose of 50.4Gy. The dose of GTV was increased by 28% in the 64.8Gy SIB-IMRT program, while the normal tissue irradiation dose was similar to that in the 50.4Gy IMRT program. In conclusion, SIB-IMRT could effectively protect normal tissues while increasing the dose of GTV in esophageal cancer from the perspective of dosimetry.

1.1.3 The innovation of radiotherapy technology could increase the fraction dose and reduce the toxicity

In recent years, hypo-fractionated radiotherapy has yielded promising results in non-small cell lung cancer and head and neck tumors [10, 11]. At present, there are few studies of hypo-fractionated radiotherapy in the treatment of esophageal cancer. A phase I/II dose-escalation study conducted by Song et al demonstrated that hypofractionated radiotherapy could improve the local control rate of esophageal cancer with a 1- and 2-year local control rate of 62% and 49%, respectively[12]. In the study, a daily dose of ≤5Gy was safe in hypofractionated radiation for the treatment of ESCC. Ma et al. Enrolled 150 patients with ESCC in a prospective randomized controlled Phase III clinical trial and compared the efficacy and toxicity of hypofractionated radiotherapy and conventionally fractionated radiotherapy. The results showed that there was no difference in 3- and 5-year overall survival rates between the

two groups. But the local failure rate of the hypo-fractionated group was significantly lower than those of the conventional fractionated group (27.0% vs 47.3%). And there was no significant difference in the incidence of toxicity between the two groups[13]. The study demonstrated that moderately hypofractionated radiotherapy might offer therapeutic benefits with improved tumor control by increasing biologically effective doses (BED).

Based on the experience of the previous study, SIB-IMRT technology was adopted in this study. Considering the tolerance of patients, a total dose of 60Gy with a fraction dose of 2.5Gy will be adopted in our study.

1.1.4 Application of S-1 in the treatment of esophageal cancer

In clinical practice, the concurrent dual-drug chemotherapeutic regimen was usually regarded as the preferred choice in the previous studies. Due to the high incidence of toxicity, its application is limited[14]. In recent years, some studies have explored new concurrent chemotherapy regimens for esophageal cancer.

S-1 is a novel, orally administered fluorouracil that is a combination of tegafur (FT), gimeracil (CDHP) and oterasil potassium. FT is the prodrug of 5-FU. CDHP competitively inhibits dihydropyrimidine dehydrogenase to maintain a high plasma concentration of 5-FU. Oterasil potassium preferentially localizes in the gut rather than in the tumor and appears to have a biochemical effect on orotate phosphoribosyltransferase thereby selectively inhibiting the formation of 5-FU nucleotides in the gut and reducing gastrointestinal side effects. In addition, S-1 can also radiosensitize tumors by inhibiting Akt/PKB activation and down-regulating thymidylate synthase [15, 16]. At present, a series of clinical studies have explored the effects of platinum-based regimens in combination with S-1 in patients with esophageal cancer. Iwasa et al, conducted a phase I prospective study involving 160 patients with LA-ESCC who received radiotherapy combined with concurrent chemotherapy with S-1 and cisplatin[17]. Radiotherapy and chemotherapy consisted of two sessions, each session of radiotherapy with a total dose of 30Gy/2Gy, combined with a 24-hour infusion of cisplatin (70mg/m2) on day 8, and oral administration of S-1 (80mg/m2/day) for two weeks. The interval of the two sessions of radiotherapy was 2 weeks. The CR rates of stage II, III and IVa patients were 91.6%, 67.6% and 36.4%, respectively. The median OS of stage II, III and IVa patients was 7.0, 2.6 and 1.3 years, respectively. The main adverse reaction was myelosuppression, and the

incidence of Grade ≥3 myelosuppression was 39%. Non-hematological toxicity including gastrointestinal reactions was mild. Another study conducted by Tsuda.T enrolled 20 patients with esophageal cancer at stage II/III. All patients received CCRT with a radiotherapy dose of ≤ 50Gy. The regimen of concurrent chemotherapy was S-1 80mg/m2 d1-14 and nedaplatin 90mg/m2 d1 every 4 weeks. The incidence of Grade 4 neutropenia, thrombocytopenia and anemia was 15%, 10% and 5%, respectively. The incidence of Grade ≥3 esophagitis and nausea was 15% and 10%, respectively. The CR rate was 80% and the 3-year OS rate was 58%[18]. Thus, S-1 as concurrent chemotherapy agents in combination with radiotherapy yielded satisfactory survival outcomes with tolerable toxicities in patients with esophageal carcinoma.

1.1.5 Summary

In conclusion, S-1 S-1 provides a more convenient way of administration and superior radiosensitizing effect compared with classical dual-drug regimen. With the innovation of the radiotherapy technology, SIB-IMRT can not only improve the dose of tumor site, but also effectively protect normal tissues. Moreover, hypofractionated radiotherapy might offer therapeutic benefits with improved tumor control. Therefore, we hypothesized that the moderately hypo-fractionated radiotherapy and concurrent S-1 would be able to reduce systemic toxicity and enhance loco-regional disease control; moreover, intensive percutaneous endoscopic gastrostomy(PEG) nutritional support and oral diet restriction during CCRT could be helpful for weight gain and reduce swallowing pain caused by radiation-induced esophagitis. We launched this prospective phase II clinical trial on the combination of moderately hypo-fractionated radiotherapy as well as concurrent S-1 and PEG nutritional support, to explore the safety and efficacy of the new potent regimen in inoperable ESCC patients.

2 Study objectives

The primary objective is to assess the safety and efficacy of the combination of moderately hypo-fractionated radiotherapy as well as concurrent S-1 and percutaneous endoscopic gastrostomy (PEG) nutritional support in inoperable LA-ESCC patients.

2.1 Primary endpoint

- PFS

2.2 Secondary endpoints

- ORR, OS, failure pattern, toxicities, nutritional status and treatment compliance

3 Investigational plan

3.1 Study design

This study is a prospective, single-arm, Phase II clinical study. Patients with LA-ESCC received hypofractionated radiotherapy once a day combined with S-1 concurrent chemotherapy. One week before CCRT, eligible patients received percutaneous endoscopic gastrostomy. The research flow chart is as follows:

Figure 3-1 Study design

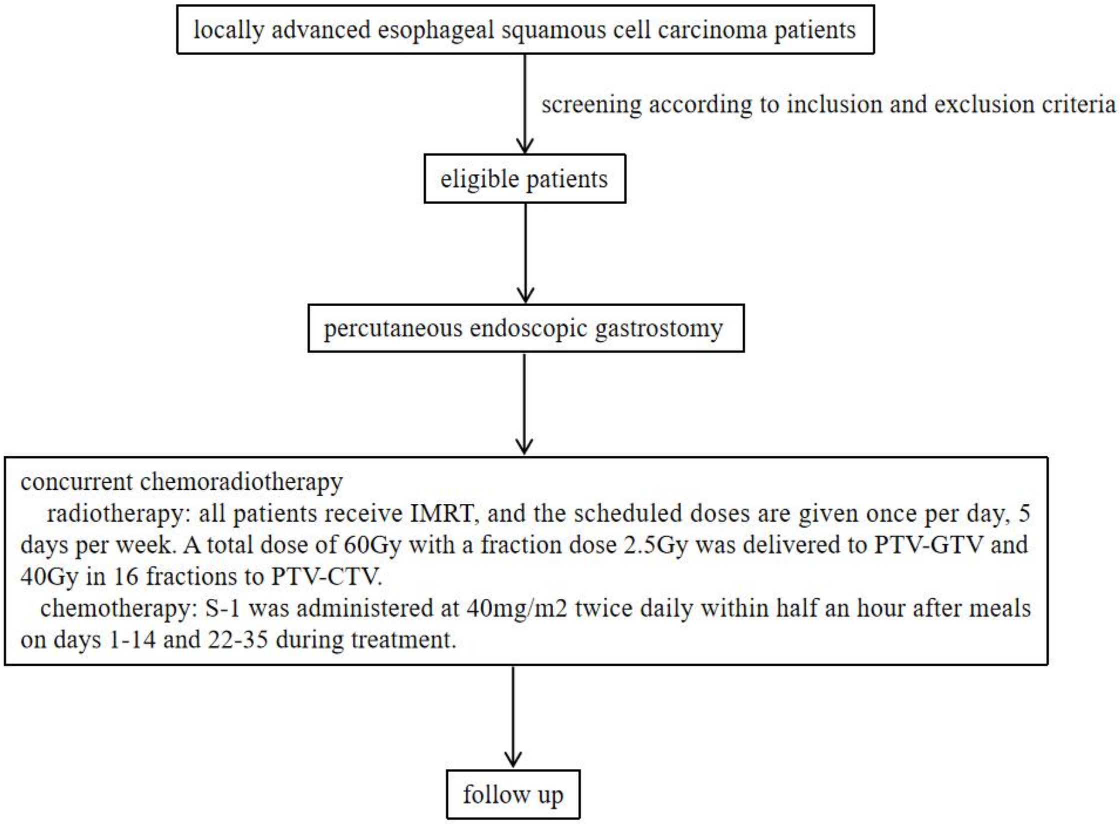

3.2 Research progress and completion time

The study is expected to be 2 years of enrollment, with all patients followed up for 3 year after completion of treatment.

4 Population

The study population will consist of male and female adult (≥ 18 years old) LA-ESCC patients diagnosed with II-IVB stages (IVB stage only with metastatic celiac or supraclavicular lymph nodes) based on the TNM staging system proposed by the International Union Against Cancer (UICC 2002).

4.1 Sample size

The Clopper-Pearson method was used to estimate the sample size. α=0.05, 1-β=0.80. The expected clinical response rate of patients with ESCC treated with radiotherapy concurrent with S-1 was 80%. Compared with the previous clinical response rate of 60%, A total of 35 subjects were required. Considering the elimination and shedding of cases during the trial, the number of cases was increased by 20% and the total of cases was determined to be ≥ 42.

4.2 Inclusion criteria

Patients eligible for inclusion in this study have to fulfill **all** of the following criteria:

1. Written informed consent must be obtained before any assessment is performed.
2. Males or females aged ≥18.
3. Histologically confirmed ESCC.
4. II-IVB stages (IVB stage only with metastatic celiac or supraclavicular lymph nodes) based on the TNM staging system proposed by the International Union Against Cancer (UICC 2002).
5. Eastern Cooperative Oncology Group (ECOG) performance status score of 0-1.
6. Charlson Comorbidity Index score≤4.
7. Capability of oral medication despite esophageal obstruction.
8. Adequate bone marrow, hepatic and renal function: white blood cell count ≥ 4 × 109/L, absolute neutrophil count ≥ 1.5 × 109/l, platelets ≥ 100 × 109/L, hemoglobin haemoglobin

≥ 100g/L, serum creatinine and bilirubin < 1.5×upper limit of normal (ULN), transaminase <2 × ULN.

4.3 Exclusion criteria

Patients fulfilling **any** of the following criteria are not eligible for inclusion in this study.

1. Contraindication for radiotherapy or chemotherapy.
2. Prior malignancies, except for curable non-melanoma skin cancer or cervical carcinoma in situ.
3. Distant metastasis, except for celiac or supraclavicular lymph nodes metastases.
4. Malignant pleual effusion or pericardial effusion.
5. Patients participating in any other clinical trial within 30 days before inclusion or concurrent with this study.
6. Uncontrollable seizures of loss of self-control due to mental illness.
7. Serious drug allergy history or allergic constitution.

4.4 Premature patient withdrawal

Patients may voluntarily withdraw from the study for any reason at any time. They may be considered withdrawn if they state an intention to withdraw, fail to return for visits, or become lost to follow-up for any other reason.

If premature withdrawal occurs for any reason, the investigator must make every effort to determine the primary reason for a patient's premature withdrawal from the study and record this information on the Study Completion Case Report/Record Form (CRF).

The investigator withdraws the subject from study under the following circumstances:

- Withdrawal of informed consent .
- Treatment cannot be performed as required by the study protocol.
- Disease progress during treatment.
- The patient is pregnant or not using adequate contraception.
- Any other protocol deviation that results in a significant risk to the patient's safety

For patients who are lost to follow-up (i.e. those patients whose status is unclear because they fail to appear for study visits without stating an intention to withdraw), the investigator should show "due diligence" by documenting in the source documents steps taken to contact the patient, e.g. dates of telephone calls, registered letters, etc.

5 Treatment

5.1 Schedule of study

Week	1	2	3	4	5
Radiotherapy	I I I I I	I I I I I	I I I I I	I I I I I	I I I I
S-1					

5.2 Concurrent chemoradiotherapy

5.2.1 Concurrent chemotherapy

S-1 was administered at 40mg/m2 twice daily within half an hour after meals on days 1-14 and 22-35 during treatment.

5.2.2 Radiotherapy

5.2.2.1 Postural immobilization

1. Postural immobilization devices should be used to ensure repeatability of patient positioning and treatment accuracy. The specific selection of immobilization devices depends on various of medical centers, but the immobilization devices used for localization must be the same as the therapeutic position.

2. Simulation CT scan is used to collect graphics, and target areas are delineated on simulated CT images, including tumor target volume (GTV), clinical target volume (CTV), planned target volume (PTV) and normal organs. The patients maintained normal shallow respirations during the CT scans. The superior margin of the scan was located at the top of C2 and the inferior border of the L4 was the inferior margin, with a thickness of 5mm.

3. In the study, a contrast-enhanced CT is used for simulation CT scan to facilitate the delineation of target areas. However, if the electron density of the enhanced tissue image is too high, it can be fused with plain CT for formulating a radiotherapy plan.

4. We encourage the use of FDG-PET/CT for simulated positioning, which must be performed

using the same postural immobilization devices as the therapeutic position. If the simulation scanning position is the same as the treatment position, the FDG-PET/CT images can be used to formulate a radiotherapy plan.

5. We encourage the use of 4D-CT scanning or the use of 4D-CT in formulating the radiotherapy plan. The following techniques could be adopted to deal with the effects of tumor and organ motion: internal target volume (ITV), maximum intensity projection (MIP), automatic breath control and respiratory gating system.

5.2.2.2 Definition of target area

The definition of radiotherapy target area follows ICRU reports 50 and 62.

1. GTV: The GTV contain the primary tumor and metastatic lymph nodes. The criteria for determing esphageal lesions on CT images are esophageal wall thickness greater than 5mm or airless esophagus greater than 10mm in diameter, esophageal localized or cicumferential thickening, and/or accompanied by local lumen stenosis. On the axial image, the length of the lesion is calculated according to the number of GTV layers outlined, and the final therapeutic target area was comprehensively determined by referring to the reference information provided by various examinations such as barium video-esophagography, contrast-enhanced computed tomography, magnetic resonance imaging (MRI) and upper gastrointestinal endoscopy. The diagnostic standard of metastatic lymph nodes is short diameter ⩾ 1.0cm. For special sites such as paraesophageal, trachesophageal groove and cardo-diaphragmatic angle, the diagnostic criteria is short diameter ⩾ 0.5cm. Mediastinal lymph nodes with standard uptake value ＞ 3 on pre-treatment PET scans should be included in the GTV.

2. CTV: The CTV consists of the primary tumor plus a 3-cm craniocaudal margin and a 1-cm circumferential margin, and metastatic lymph node plus a 0.5- to 1-cm expansion margin. For lower-thoracic ESCC, the paracardial, lesser gastric curvature and the left gastric artery nodes were included in CTV, while for cervical and upper-thoracic ESCC, the supraclavicular area was included.

3. PTV: The external boundary of PTV needs to consider the internal motion and set-up margin of internal organs. In the study, the PTV was derived from expanding GTV and CTV with a 0.5cm margin in all directions, respectively.

4. It is recommended that image-guided technology (KV-X-ray online guidance, Cone-beam CT online or offline guidance) should be to quantify, correct and record the positioning errors.

5. The delineation of the target area and normal tissue should be carried out by the radiotherapy physicians, and it is recommended to improve the accuracy of the delineation of the target area through the consultation of the radiologist.

5.2.2.3 Radiotherapy plan

In the study, intensity-modulated radiation therapy (IMRT) technology is applied to formulate a radiotherapy plan.

5.2.2.4 Dosage prescription

All patients receive IMRT, and the scheduled doses are given once per day, 5 days per week. A total dose of 60Gy with a fraction dose 2.5Gy was delivered to PTV-GTV and 40Gy in 16 fractions to PTV-CTV. And at least 95% of PTV volume received 95% of the prescription dose. The maximum dose of GTV does not exceed 68 Gy. The minimum dose of PTV cannot be less than 95% of the prescribed dose. The dose calculation should take into account the difference the difference in tissue density in the irradiated area (ie, air density of lung and bone) to correct for tissue heterogeneity.

The maximum dose point and the minimum dose point with the PTV should recorded. The dose distribution inside and outside the PTV must be evaluated, and dose deviations distinguished and adjusted accordingly.

- No deviation: Greater than or equal to 99% of the PTV volume receives at least 95% of the prescribed dose. No area ≥$1cm^3$ in the PTV receives >110% of the prescribed dose, and no adjacent $1cm^3$ area outside the PTV receives >110% of the prescribed dose.
- Slight deviation: This deviation is acceptable but should be avoided. Greater than or equal to 95% of PTV volume but less than or equal to 99% of PTV volume receives at least 95% of the prescribed dose. An area with volume > $1cm^3$ within the PTV receives greater than 110% but less than or equal to 115% of the prescribed dose, and an adjacent area with volume ≤$1cm^3$ outside the PTV receives greater than 110% but less than or equal to 115% of the prescribed dose.

- Serious deviation: This deviation is unacceptable. Less than 95% of the PTV volume receives at least 95% of the prescribed dose. An area with volume ＞1cm^3 within the PTV receives greater than 115% of the prescribed dose, and an adjacent area with volume ⩾1cm^3 outside the PTV receives greater than 115% of the prescribed dose.

5.2.2.5 Organ at risk

Normal tissue tolerance should be fully considered when formulating a radiotherapy plan. When the target dose coverage is difficult to balance with the dose limit of organs at risk, the dose limit of organs at risk is preferred.

- Spinal cord: Spinal canal shape is outlined to represent the spinal cord. The Dmax of the spinal cord is 46Gy. The dose of the spinal cord over 50Gy is considered a serious deviation.
- Lungs: The V20 of the lungs (except the target area) should not exceed 30%. The V5 of the lungs (except target area) should not exceed 70%, and the mean lung dose (MLD) does not exceed 13Gy.
- Heart: The upper boundary of delineation of heart should include the infundibular portion of the right ventricle and the tops of both atria, but the large vessels should be removed as much as possible. The lower boundary should be the lowest point of the left ventricle, which should be separated from the liver. The V30 of the heart should not exceed 30%.
- Liver: The V30 of the liver should not exceed 20%.

5.2.2.6 Delivery of treatment

X-ray with energy ⩾6 MV is used for treatment. In the study, we recommend the use of a dynamic multi-leaf linear accelerator, avoiding the use of cyberknife, gamma knife and Tomotherapy. Try to ensure that the enrolled patients receive radiotherapy by the same treatment planning system and the same linear accelerator, and try to complete treatment in the same linear accelerator for each patient (except for forcing majeure external reasons such as long-term mechanical failure).

5.2.2.7 Quality control

Prior to enrolling the first patient, the study center will be evaluated using a simulated case.

The radiotherapy plan for the simulated case is reviewed to ensure that the radiotherapy prescription meets protocol requirements. The positioning and radiotherapy plan for each enrolled patient will be reviewed after enrollment.

The schedule quality certification (annual, monthly, weekly, daily) of simulation CT, treatment planning system and linear accelerator will be reviewed before enrollment.

It is required that the electronic documentation of the radiotherapy plan for each enrolled patient must be backed up and archived. Dose-volume histogram (DVH) should describe the target area, the lungs, the lungs, the spinal cord, and the heart. The following dose values should be recorded: the dose at the point of prescription, the minimum dose, the maximum dose and the average dose of the PTV, the maximum dose of the spinal cord, the V20 and V5 of the lungs, the average dose and the maximum dose of the heart.

5.3 Percutaneous endoscopic gastrostomy (PEG)

One week before CCRT, eligible patients receive percutaneous endoscopic gastrostomy. Gastrostomy tube is placed using the “pull“ method introduced by Ponsky et al. In this method, a thin string is inserted into the stomach through a needle in the abdominal wall, grabbed with endoscopic biopsy forceps and then taken out through the esophagus and mouth. Subsequently, the cord is fixed to the outer end of the gastrostomy tube and the tube is pulled from the mouth to the esophagus, stomach, and out through the abdominal wall. Proton pump inhibitors and antibiotics are routinely used for 3 days after PEG. Subsequently, patients need to follow a strict oral diet protocol: 1)all nutritional supplements are given via gastrostomy tube during and 2-3 months post-CCRT; 2) Only saline solution, purified water and S-1 are allowed to be taken orally before gastrostomy tube removal; 3) oral liquid diet could be recovered when endoscopy shows no ulcer or residual tumor, PG-SGA score $<$ 4 points, weight loss less than 2% within 1 month and the nutrition-related indicators meeting standard levels (hemoglobin $\geqslant$ 100g/L, albumin $\geqslant$ 35g/L and prealbumin $\geqslant$ 20mg/mL); 4) It is recommended to retain the gastrostomy tube for 1-2 months after resuming oral liquid diet, and then removed the tube if there are no abnormalities.

6 Adverse events and treatment plan adjustment

All treatment-related acute and late toxicities were graded in terms of the Common Terminology Criteria for Adverse Events (CTCAE) of the National Cancer Institute (version 4.0) and record on the Study Completion CRF. Serious adverse events (SAE) must be reported to the institutional review board within 24h and dealt with properly.

6.1 Definition

6.1.1 Adverse events (AE)

1. An adverse event (AE) is any untoward medical occurrence (i.e., any unfavorable and unintended sign [including abnormal laboratory findings], symptom or disease) in a subject or clinical investigation subject *after providing written informed consent* for participation in the study.

2. The occurrence of adverse events should be sought by non-directive questioning of the patient at each visit during the study. Adverse events also may be detected when they are volunteered by the patient during or between visits or through physical examination, laboratory test, or other assessments.

3. Abnormal laboratory values or test results constitute adverse events only if they fulfill at least one of the following criteria:

- they induce clinical signs or symptoms
- they are considered clinically significant
- they require therapy

4. Clinically significant abnormal laboratory values or test results should be identified through a review of values outside of normal ranges/clinically notable ranges, significant changes from baseline or the previous visit, or values which are considered to be non-typical in patient with underlying disease. Investigators have the responsibility for managing the safety of individual patient and identifying adverse events.

5. Adverse events should be recorded in the Adverse Events CRF under the signs, symptoms or diagnosis associated with them accompanied by the following information

- the severity grade

- its relationship to the previous study treatment
- its duration (start and end dates) or if the event is ongoing an outcome of not recovered/not resolved should be reported.
- whether it constitutes a serious adverse event (SAE)
- whether other medication or therapies have been taken (concomitant medication/non-drug therapy)
- its outcome (not recovered/not resolved; recovered/resolved; recovering/resolving, recovered/resolved with sequelae; fatal; or unknown)

6.1.2 Serious adverse events (SAE)

An SAE is any adverse event (appearance of (or worsening of any pre-existing) undesirable sign(s), symptom(s) or medical conditions(s) which meets any one of the following criteria:

- is fatal or life-threatening.
- results in persistent or significant disability/incapacity.
- constitutes a congenital anomaly/birth defect
- requires inpatient hospitalization or prolongation of existing hospitalization
- is medically significant, i.e. defined as an event that jeopardizes the patient or may require medical or surgical intervention to prevent one of the outcomes listed above.

6.2 Toxicity of radiotherapy and management of radiation-related side effects

1. Interruption of radiotherapy should be avoided as far as possible.

2. Hematologic toxicity: In the event of nerutropenia with fever, confirmed infection or bleeding, radiotherapy is interrupted until the toxicity degrades to Grade ≤ 2, and the interruption time of radiotherapy should be ≤ 14 days.

3. If radiotherapy is interrupted due to treatment-related toxicities, S-1 should be discontinued accordingly. In order to ensure the smooth implementation of radiotherapy, appropriate preventive measures and nutritional support can be used. In rare cases, if patients have side effects unrelated to the treatment or unforeseen, the investigator should carefully decide whether the treatment needs to be discontinued. Treatment can be continued after the toxicity

degrades to Grade ≤ 2, and the interruption time of radiotherapy should be ≤ 14 days.

4. The treatment of radiation pneumonitis and esophagitis can refer to the treatment methods in the following table

	Adverse events (CTCAE 4.0)	Treatment
Radiation esophagitis	Grade 1 (Asymptomatic, clinical or diagnostic observation only)	observation
	Grade 2 (Symptomatic, altered eating/swallowing, intravenous nutritional support < 24 hours)	symptomatic treatment
	Grade 3 (Symptomatic, severe dietary/swallowing changes, inadequate intake of calories or fluids through the mouth, need for intravenous nutritional support, gastric tube feeding, total parenteral nutrition > 24h)	Lidocaine gel + calcium carbonate or antacid + sucralfate<br>Nutritional support, medication, nasogastric catheterization or gastrostomy if necessary
	Grade 4 (Life-threatening consequences)	Ranitidine, or other H-2 blockers or proton pump inhibitors<br>Nasogastric catheterization or gastrostomy<br>Interruption radiotherapy and/or S-1
Radiation pneumonitis	Grade 1 (Asymptomatic, clinical or diagnostic observation only)	observation
	Grade 2 (Symptomatic, alterations in lung function can not hinder quality of life and performance in activities of daily living.)	symptomatic treatment
	Grade 3 (Symptomatic, alterations in lung function hinder the quality of life and performance in activities of daily living)	Anti-infective: the causative organism should be identified as soon as possible and antibiotics should be selected according to the susceptibility test<br>Interruption radiotherapy and/or S-1
	Grade 4 (Life-threatening respiratory compromise)	Adrenocortical hormones<br>Interruption radiotherapy and/or S-1<br>urgent intervention indicated (e.g., tracheotomy or intubation

6.3 Toxicity of chemotherapy and adjustment of chemotherapy regimen

6.3.1 Common adverse events of chemotherapy

The common toxicity of S-1 monotherapy include leukopenia, thrombocytopenia, and gastrointestinal

reactions.

6.3.2 Chemotherapy dose adjustment

1. All patients are given chemotherapy doses according to the protocol, which could be adjusted as necessary for the most severe hematological or other toxicity. Any patient who requires a dose reduction will continue to receive the reduced dose for a subsequent treatment cycle. If multiple toxicities occur in the patient and dose adjustment principles differ from one to the other, the minimum dose is selected.

1. In the event of Grade 2 thrombocytopenia, anemia, hepatic or renal dysfunction, Grade 3 leukopenia/neutropenia, Grade 2 radiation esophagitis, pneumonitis and other Grade 2 non-hematological toxicities, the dosage of S-1 was reduced by 25%. If more severe toxicity occurred S-1 was suspended. However, if the adverse events degraded to Grade 0-1 within 1 week of drug withdrawal, the patient could retake S-1 at 75% of the original dose, otherwise, S-1 was terminated henceforward.

7 Combined medication

7.1 Prophylactic medication

Prophylactic antiemetic therapy with a 5-HT3 antagonist such as ondansetron, granisetron, or dorasetron is given during each cycle of chemotherapy.

7.2 Concomitant medication

7.2.1 Supportive care

Nutritional support and immune-supportive treatment should be provided as needed, and measures taken should be documented in the case report form.

7.2.2 Other drugs

- Antibiotics.
- Antiallergic.
- G-CSF, GM-CSF and other growth factors.

8 Visit schedule and assessments

8.1 The baseline assessment

All patients must complete the following required tests before being considered for inclusion in the stduy, The baseline assessment is generally required to be completed with 21 days before treatment.

Subjects		Time
Informed consent		Prior to treatment
Medical history & physical examination	Medical history: history of concomitant disease and medication.<br>Physical examination: height, weight, Body Mass Index (BMI), ECOG score, Charlson Complication Index score, patient-generated subjective global assessment (PG-SGA) score, and systemic examination	Within 14 days prior to treatment
Routine examination	Blood routine, urine routine, stool routine, blood type and coagulation function	Within 14 days prior to treatment
Serological tests	Blood glucose, blood lipid, liver and kidney function, albumin, prealbumin, electrolyte	Within 14 days prior to treatment
Imaging examinations	a. CT examination of the lower neck, chest and upper abdomen, the scanning range from the 4th cervical spine to the umbilical plane, and the routine enhanced scan is performed after the plain scan.<br>b. MRI examination of the lower neck and chest, the scanning range is from the 4th cervical spine to the diaphragm plane, and the routine enhanced scan plus diffuse imaging is performed after plain scan.<br>c. Barium video-esophagography;<br>d. Bone scan, optional if necessary to exclude bone metastases<br>e. PET-CT, optional if needed to exclude distant metastases	Within 21 days prior to treatment
Electrocardiogram	12-lead electrocardiogram	Within 21 days prior to treatment
Cardiac ultrasound	Assessment of ejection fraction and heart valves lesions	Within 21 days prior to treatment
Pulmonary function test	Pulmonary function test and blood gas analyses	Within 21 days prior to treatment
Pathological examination	upper gastrointestinal endoscopy and biopsy	Within 21 days prior to treatment
Other examinations based on clinical need		

8.2 The assessment during the treatment

Subjects		Time
Medical history & physical examination	Medical history: concomitant medication. Physical examination: height, weight, BMI, ECOG score, PG-SGA score, and systemic examination	Once a week
Routine examination	Blood routine, urine routine, stool routine	Once a week
Serological tests	Blood glucose, blood lipid, liver and kidney function, albumin, prealbumin, electrolyte	Once a week
Imaging examinations	Barium video-esophagography and chest radiography	Twice a week
Electrocardiogram	12-lead electrocardiogram	If necessary
Other examinations based on clinical need		

8.3 The efficacy assessment at two months after the completion of treatment

The following assessment should be completed at two months after the completion of treatment:

- Medical history & physical examination: history of concomitant disease and medication, height, weight, BMI, ECOG score, PG-SGA score, and systemic examination
- Routine examination: blood routine, urine routine, stool routine, and coagulation function
- Serological tests: Blood glucose, blood lipid, liver and kidney function, albumin, prealbumin, and electrolyte
- Electrocardiogram
- Contrast-enhanced computed tomography (CT) of neck, chest and abdomen: the scanning range from the 4th cervical spine to the umbilical plane.
- Contrast-enhanced magnetic resonance imaging(MRI) of the lower neck and chest: the scanning range is from the 4th cervical spine to the diaphragm plane.
- Upper gastrointestinal endoscopy and biopsy (if necessary).

8.4 Quality of life score

The quality of life of the patients is assessed using the EORTC Quality of Life Measurement

Scale QLQ-C30 before treatment, 3 weeks after the intiation of treatment, 6 weeks after the intiation of treatment, and 2 monthe after the completion of treatment.

8.5 Follow up

All patients are followed every 3 months for the first 2 years, every 6 months for years 3-5 and yearly thereafter. Every follow-up include history, physical examination, blood routine, basic metabolic panel, contrast-enhanced CT of the neck, thorax and abdomen, contrast-enhanced chest MRI, endoscopy and biopsy (if necessary). Notably, endoscopy should be routinely performed at each post-treatment follow-up in the study.

9 Evaluation of the research results

9.1 Efficacy

9.1.1 Evaluation criteria for the treatment response

The Response Evaluation Criteria in Solid Tumors (RECIST) is applied to classify tumor response:

- Complete response (CR): All target lesions disappear. (1) Barium video-esophagography: 1) the lesions disappear, 2) the walls of the esophagus are soft, 3) barium passes smoothly. (2) endoscopy and biopsy: no residual tumor. (3) CT imaging: 1) no obvious thickening of the esophageal wall, 2) the thickness of the esophageal wall $<$ 5mm, 3) the metastatic lymph nodes disappear, 4) no new lesions appear. All of the above are maintained for more than 4 weeks.
- Partial response (PR): Based on barium video-esophagography and CT imaging, the sum of the longest diameter of all baseline target lesions is reduced by $\geq$30% and maintained for 4 weeks.
- Stable disease (SD): The sum of the longest diameter of all baseline target lesions has neither sufficient shrinkage to qualify for PR nor sufficient increase to qualify for PD.
- Progressive disease (PD): Based on barium video-esophagography and CT imaging, the sum of the longest diameter of all baseline target lesions increases by $\geq$20%, or one or more lesions appear.

9.1.2 Objective response rate

The objective response rate (ORR) is defined as the rate of complete response and partial response.

9.1.3 Survival outcomes

1. Progression-free survival (PFS): PFS is calculated from treatment start to the date of locoregional failure or distant metastasis or death, whichever occurs first.

2. Overall survival (OS): OS is calculated from treatment start to the date of death from any cause or censored at the last follow-up.

9.1.4 Nutritional status

From the initiation of the treatment to 6 months post-CCRT, the patient-generated subjective global assessment (PG-SGA) score and nutrition-related indicators including weight, total protein, albumin, prealbumin, hemoglobin and lymphocyte were monitored and recorded regularly.

9.1.5 Failure patterns

1. Locoregional failure: It is defined as intraesophageal recurrence and/or the mediastinum, supraclavicular and scalenus region, left gastric and cardia region, or other lymph node drainage area metastasis.

2. Distant metastasis

9.2 Safety assessment

9.2.1 Overall safety

With the exception of nausea and vomiting, all toxicities observed within 24 hours of administration should be recorded. The severity grade of toxicities and its relationship to the study drug should be carefully analyzed.

9.2.2 Clinical safety

The safety assessment should include all subjects in each treatment cycle. The following tests should be performed on the designated dates before, during, and after treatment:

- Medical History of malignant or non-malignant disease.
- Toxicity and symptoms due to previous treatment should be assessed based on

adequate clinical examination and physical examination

Adverse events/symptoms: Adverse events and symptoms associated with the disease should be regularly assessed according to NCI CTCAE version 4.0.

9.2.3 Laboratory evaluations

The change in hematology, chemistry, prothrombin time, and nutrition-related indicators will be assessed at all visits as an overall measure of safety over time.

Blood samples for hematology and biochemistry evaluations are to be taken preferably after a 4 hours fast.

9.2.3.1 Hematology

Red blood cell (RBC) count, haemoglobin (Hb), haematocrit (Ht), mean corpuscular volume (MCV), mean corpuscular hemoglobin concentration (MCHC), WBC count, differential WBC count, and platelet count will be measured.

9.2.3.2 Biochemistry

Electrolytes (sodium, potassium, calcium, chloride, magnesium, and phosphate), albumin, prealbumin, total protein, glucose, creatinine, calculated creatinine clearance, blood urea nitrogen (BUN), uric acid, alkaline phosphatase, ALT, AST, total and conjugated bilirubin, bile acids, gamma glutamyl transferase (γ-GT), LDH, amylase, lipase, cholesterol (total and HDL), triglycerides, vitamin D (1, 25 Dihydroxy Vitamin D3), TSH, free T3 and T4 will be measured.

9.2.3.3 Coagulation testing

Prothrombin time (PT expressed as INR) and fibrinogen will be measured.

9.2.4 Appropriateness of safety measurements

The safety assessments selected are standard for this indication/patient population.

10 Statistics

10.1 Analysis sets

1. Intent-to-treat population (ITT): The ITT set consists of all subjects who signed informed consent and were included regardless of whether they completed the entire treatment process. ITT sets are used for safety and efficacy analysis.

2. Per-protocol population (PP): The PP set is defined as a subgroup of ITT set. PP set refers to those who meet the conditions of ITT set and complete all treatment as per protocol. It is generally used for the primary efficacy analysis.

10.2 Patient demographics and other baseline characteristics

Summary statistics will be provided for patient demographics (age, sex, race, ethnicity, height, weight and BMI) and other baseline disease characteristics such as PG-SGA score. Continuous variables will be presented with mean, median, 25th percentile, 75th percentile, standard deviation, minimum and maximum, and the number of non-missing observations. Categorical data will be displayed via absolute and relative frequencies for each category (including a category labeled as 'missing' when appropriate).

10.3 Analysis of the primary and key secondary variables

10.3.1 Variables

The primary efficacy variable is PFS. The key secondary variables are ORR, OS, failure pattern, toxicities, nutritional status and treatment compliance

10.3.2 Statistical model and method of analysis

Descriptive statistics are conducted for the analysis of baseline characteristics and treatment-related toxicities. All data analyses are conducted using SPSS version 22.0. The survival rates are estimated using the Kaplan-Meier method and differences in the survival curves are compared by the log-rank test. Statistical comparisons are performed by using unpaired t-tests. $P<0.05$ is considered statistically significant.

11 Ethical considerations

11.1 Regulatory and ethical compliance

This clinical study was designed and shall be implemented and reported in accordance with the International Conference on Harmonization (ICH) Harmonized Tripartite Guidelines for Good Clinical Practice, with applicable local regulations , and with the ethical principles laid down in the Declaration of Helsinki.

11.2 Informed consent procedures

Eligible patients may only be included in the study after providing written (witnessed, where required by law or regulation), IRB/IEC-approved informed consent, or, if incapable of doing so, after such consent has been provided by a legally acceptable representative of the patient. In cases where the patient's representative gives consent, the patient should be informed about the study to the extent possible given his/her understanding. If the patient is capable of doing so, he/she should indicate assent by personally signing and dating the written informed consent document or a separate assent form. Informed consent must be obtained before conducting any study-specific procedures (i.e. all of the procedures described in the protocol). The process of obtaining informed consent should be documented in the patient source documents.

11.3 Responsibilities of the investigator and IRB/IEC

The protocol and the proposed informed consent form must be reviewed and approved by a properly constituted Institutional Review Board/Independent Ethics Committee/Research Ethics Board (IRB/IEC) before study start. Prior to study start, the investigator is required to sign a protocol signature page confirming his/her agreement to conduct the study in accordance with these documents and all.

11.4 Publication of study protocol and results

Upon study completion and finalization of the study report the results of this trial will be either submitted for publication and/or posted in a publicly accessible database of clinical trial results.

12 Protocol adherence

Investigators ascertain they will apply due diligence to avoid protocol deviations.

This protocol defines the study objectives, the study procedures and the data to be collected on study participants. Under no circumstances should an investigator collect additional data or conduct any additional procedures for any research related purpose involving any investigational drugs.

If the investigator feels a protocol deviation would improve the conduct of the study this must be considered a protocol amendment, and unless such an amendment is approved by the

IRB/IEC it cannot be implemented. All significant protocol deviations will be recorded and reported in the CSR.

12.1 Protocol amendments

Any change or addition to the protocol can only be made in a written protocol amendment that must be approved by the IRB/IEC. Only amendments that are required for patient safety may be implemented prior to IRB/IEC approval. Notwithstanding the need for approval of formal protocol amendments, the investigator is expected to take any immediate action required for the safety of any patient included in this study, even if this action represents a deviation from the protocol. In such cases, the IRB/IEC at the study site should be informed within 10 working days.

13 Reference

1. Lin Y, Totsuka Y, He Y, et al. Epidemiology of esophageal cancer in Japan and China. J Epidemiol 2013;23:233–42.

2. Cooper JS, Guo MD, Herskovic A, et al. Chemoradiotherapy of locally advanced esophageal cancer: long-term follow-up of a prospective randomized trial (RTOG 85-01). Radiation Therapy Oncology Group. JAMA. 1999 May 5;281(17):1623-7.

3. Minsky BD, Pajak TF, Ginsberg RJ, et al. INT 0123 (Radiation Therapy Oncology Group 94-05) phase III trial of combined-modality therapy for esophageal cancer: high-dose versus standard-dose radiation therapy. J Clin Oncol. 2002 Mar 1;20(5):1167-74.

4. Gaspar LE, Winter K, Kocha WI, et al: A phase I/II study of external beam radiation, brachytherapy, and concurrent chemotherapy for patients with localized carcinoma of the esophagus (Radiation Therapy Oncology Group Study 9207): final report. Cancer.2000;88:988-95.

5. Fan TY, Xing J, Lu J, et al. Phase I/II study of induction chemotherapy plus concurrent chemotherapy and SMART-IMRT-based radiotherapy in locoregionally-advanced nasopharyngeal cancer. Oncol Lett. 2013;5(3):889-95.

6. Koom WS, Kim TH, Shin KH, et al. SMART (simultaneous modulated accelerated radiotherapy) for locally advanced nasopharyngeal carcinomas. Head Neck. 2008;30(2):159-69.

7. Butler EB, Teh BS, Grant WH 3rd, et al. Smart (simultaneous modulated accelerated radiation therapy) boost: a new accelerated fractionation schedule for the treatment of head and neck cancer with intensity

modulated radiotherapy. Int J Radiat Oncol Biol Phys. 1999;45(1):21-32.

8. Settle SH, Bucci MK, Palmer MB, et al. PET/CT fusion with treatment planning CT (TP CT) shows predominant pattern of locoregional failure in esophageal patients treated with chemoradiation(CRT) is in GTV. Int J Radiat Oncol Biol Phys.2008;72:S72–S73.

9. Welsh J, Palmer MB, Ajani JA, et al. Esophageal cancer dose escalation using a simultaneous integrated boost technique. Int J Radiat Oncol Biol Phys. 2012 Jan 1;82(1):468-74. doi: 10.1016/j.ijrobp.2010.10.023. Epub 2010 Dec 1.

10. Porceddu SV, Rosser B, Burmeister BH, et al. Hypofractionated radiotherapy for the palliation of advanced head and neck cancer in patients unsuitable for curative treatment--"Hypo Trial". Radiother Oncol.2007;85:456-62.

11. Agarwal JP, Nemade B, Murthy V, et al. Hypofractionated, palliative radiotherapy for advanced head and neck cancer. Radiother Oncol.2008;89:51-6.

12. Song YP, Ma JB, Hu LK, et al. Phase I/II study of hypofractioned radiation with three-dimensional conformal radiotherapy for clinical T3-4N0-1M0 stage esophageal carcinoma. Technol Cancer Res Treat.2011;10:25-30.

13. Ma JB, Wei L, Chen EC, et al. Moderately hypofractionated conformal radiation treatment of thoracic esophageal carcinoma. Asian Pac J Cancer Prev.2012;13:4163-7.

14. Tepper J, Krasna MJ, Niedzwiecki D, et al. Phase III trial of trimodality therapy with cisplatin, fluorouracil, radiotherapy, and surgery compared with surgery alone for esophageal cancer: CALGB 9781. J Clin Oncol.2008;26:1086-92.

15. Harada K, Kawaguchi S, Supriatno H, et al. S-1, an oral fluoropyrimidine anti-cancer agent,enhanced radiosensitivity in a human oral cancer cell line in vivoand in vitro: involvement possibility of inhibition of survival signal,Akt/PKB. Cancer Lett 226:161-8.

16. N akata E, Fukushima M, Takai Y et al (2006) S-1, an oral fluoropyrimidine,enhances radiation response of DLD-1/FU humancolon cancer xenografts resistant to 5-FU. Oncol Rep 16:465–71.

17. Iwase H, Shimada M, Tsuzuki T, et al. Concurrent chemoradiotherapy with a novel fluoropyrimidine, S-1, and cisplatin for locally advanced esophageal cancer: long-term results of a phase II trial. Oncology.2013;84:342-9.

18. Takashi Tsuda, Hiroyuki Inaba, et al. Prospective study of definitive chemoradiotherapy with S-1 and nedaplatin in patients with stage II/III (non-T4) esophageal cancer. Esophagus March 2011, Volume 8, Issue 1, pp 45-51
